# Long time behavior and stable patterns in high-dimensional polarity models of asymmetric cell division

**DOI:** 10.1007/s00285-021-01619-w

**Published:** 2021-06-07

**Authors:** Yoshihisa Morita, Sungrim Seirin-Lee

**Affiliations:** 1 Department of Applied Mathematics and Informatics, Ryukoku University, Seta, Otsu 520-2194 Japan; 2 Department of Mathematics, Department of Mathematical and Life Sciences, Graduate School of Integrated Science for Life, Hiroshima University, Kagamiyama 1-3-1, Hiroshima, 700-0046 Japan

**Keywords:** ., 35B36, 35B40, 35K57, 92C15, 92C37

## Abstract

Asymmetric cell division is one of the fundamental processes to create cell diversity in the early stage of embryonic development. During this process, the polarity formation in the cell membrane has been considered as a key process by which the entire polarity formation in the cytosol is controlled, and it has been extensively studied in both experiments and mathematical models. Nonetheless, a mathematically rigorous analysis of the polarity formation in the asymmetric cell division has been little explored, particularly for bulk-surface models. In this article, we deal with polarity models proposed for describing the PAR polarity formation in the asymmetric cell division of a *C. elegans* embryo. Using a simpler but mathematically consistent model, we exhibit the long time behavior of the polarity formation of a bulk-surface cell. Moreover, we mathematically prove the existence of stable polarity solutions of the model equation in an arbitrary high-dimensional domain and analyse how the boundary position of polarity domain is determined. Our results propose that the existence and dynamics of the polarity in the asymmetric cell division can be understood universally in terms of basic mathematical structures.

## Introduction

**Fig. 1:**
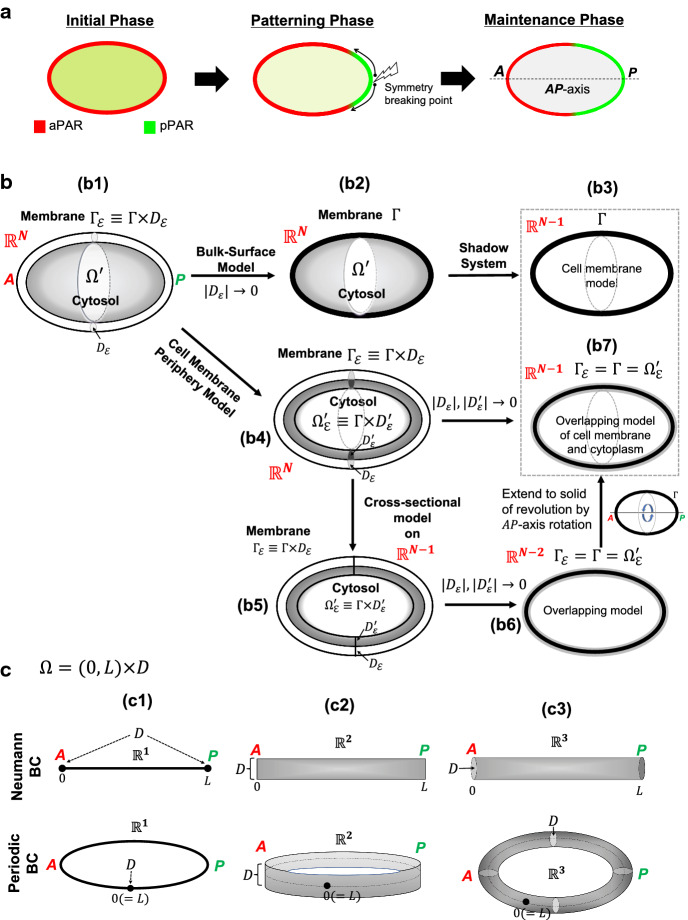
PARs polarity in asymmetric cell division and schematic diagram of model. **a** PAR polarity process in a *C. elegans* embryo is divided into three phases: symmetry breaking during the initial phase, the patterning(establishment) phase of an emerging pattern, and the maintenance phase of the stationary state of polarity. *** A, P*** indicate the points of anterior and posterior poles, respectively. **b** Schematic diagram of model reductions is shown. Gray-coloured regions imply cytoplasm and black-colored thick line region implies cell membrane. $D_{\varepsilon }$ and $D_{\varepsilon }^{'}$ are the cross-sectional areas of membrane and cytoplasmic spaces which are separated by the inner boundary region ($\Gamma$) between cell membrane and cytosol. $\Omega '$ indicates bulk cytosol space in $\mathbb {R}^N$, and $\Omega {'}_{\varepsilon }$ is the cytosol region around the cell membrane region, $\Gamma ^{'}_{\varepsilon }$. **c** The domain shapes of $\Omega =(0, L)\times D$ are shown with respect to Neumann and periodic boundary conditions. *D* is defined by the edge points of line, the vertical line, and cross-sectional area in one, two, and three dimensional spaces, respectively (color figure online)

The polarity formation emerging during the early stage of embryonic development is a spectacular mechanism wherein a single mother cell of fertilised egg creates completely different daughter cells through asymmetric cell divisions (Campanale et al. 2017; Gönczy 2005). Because the polarity formation has initial and central roles during the entire process of asymmetric cell divisions, an elucidation of the polarity mechanism has been extensively explored in both experimental and theoretical approaches (see the recent review papers; Lang and Munro 2017; Rappel and Edelstein-Keshet 2017; Cortes et al. 2018). In particular, the PAR polarity formation in a *C. elegans* embryo has been well studied as one of a representative biological model. In the asymmetric cell division of *C. elegans* embryo, anterior PARs (aPAR) and posterior PARs (pPAR) are exclusively formed in an asymmetrical manner, which plays a key role in redistributing the cytosol substrates (Fig. 1a). PAR proteins are mostly upstream regulators that control the downstream proteins and a series of the processes of asymmetric cell division (Cuenca et al. 2002; Hoege and Hyman 2013; Wu et al. 2018). During the initial stage, aPAR and pPAR are homogeneously distributed in the membrane and cytosol, respectively. After the symmetry is broken by sperm entry, the pPAR spreads from the posterior pole, and the growth of the domain of pPAR polarity stops at approximately half the egg length with the formation of the two segregated polarity domains of aPAR and pPAR. This is consequently maintained for approximately 16 min, and a mother cell prepares for cell division (Gönczy 2005).

Owing to the key role of PAR polarity in asymmetric cell division, several mathematical models for the PAR polarity formation have been suggested and well-explored in recent years (Cortes et al. 2018; Goehring et al. 2011b; Marée et al. 2006; Seirin-Lee and Shibata 2015; Seirin-Lee 2016b, 2020; Trong et al. 2014). Although the details of their model descriptions are different, a mathematical structure that generates a polarity pattern is taken into account universally, and the bi-stability structure in the kinetic terms and the property of mass conservation have been noted as the basal conditions. Nonetheless, few studies on the PAR polarity in terms of high-dimensional models reflecting the cell geometry of a bulk cytosol space and a surface cell membrane have been conducted numerically or mathematically. In particular, a rigorous mathematical analysis of the PAR polarity formation has been poorly explored, and most of the previous studies have been based on numerical observations. Although some mathematical studies have been conducted on PAR polarity models, they have dealt with one-dimensional problems or reduced systems with less variables (Kuwamura et al. 2018; Morita and Ogawa 2010; Morita 2012; Seirin-Lee et al. 2020). Moreover, the most fundamental question for the existence and stability of polarity solutions with two exclusive polarity domains has been infrequently studied owing to mathematical difficulties. Here, related to bulk cytosol-surface models, we refer to studies on Turing-type instability (Levine and Rappel 2005; Morita and Sakamoto 2018, 2020; Rätz and Röger 2012, 2014), the existence and stability of a polarized solution reduced on the sphere (Diegmiller et al. 2018), and polarized patterns numerically shown in 3-dimensional domains with complex geometries (Cusseddu et al. 2019), where different types of reaction–diffusion systems are investigated. The readers who are interested in a basic mathematical theory may refer to Sharma and Morgan (2016) for the existence of time global solutions.

In this article, we consider the aPAR-pPAR polarity models suggested by Seirin-Lee and Shibata (2015) and Goehring et al. (2011b), and extend these models to include a high-dimensional case with a cell geometry composed of a bulk cytosol space and a cell membrane. The cell membrane consists of a lipid bilayer and contains membrane proteins which diffuse laterally through the membrane (Cooper 2000). Because a typical eukaryotic cell has a cell membrane thickness of 5–10 nm compared to a cell diameter of about 50 $\upmu$m, the cell membrane can be considered as a surface domain with thin thickness (Fig. 1b(b1)). Then, the model system is described as follows:

1.1 $$\begin{aligned} \begin{aligned}&\partial _t{P_m}=D_m\Delta {P_m} -F_{\text {off}}(A_m){P_m}+\gamma {P_c}\quad \mathrm {in} \quad \Gamma _{\varepsilon }, \\&\partial _t{P_c}=D_c\Delta {P_c}\quad \mathrm {in}\quad \Omega ', \\&D_c\partial _\nu {P_c}=F_{\text {off}}(A_m){P_m}-\gamma {P_c}\quad \mathrm {on}\quad \Gamma , \\&\partial _t{A_m}=\overline{D}_m\Delta {A_m} -\overline{F}_{\text {off}}(P_m){A_m}+{\overline{\gamma }}{A_c}\quad \mathrm {in} \quad \Gamma _{\varepsilon }, \\&\partial _t{A_c}=\overline{D}_c\Delta {A_c}\quad \mathrm {in}\quad \Omega ', \\&D_c\partial _\nu {A_c}=\overline{F}_{\text {off}}(P_m){A_m}-{\overline{\gamma }}{A_c}\quad \mathrm {on}\quad \Gamma , \end{aligned} \end{aligned}$$

where $\Omega '$ is a bulk cytosol and $\Gamma _{\varepsilon }=\Gamma \times D_{\varepsilon }$. $\Gamma (=\partial \Omega ')$ is the surface of a bulk cytosol space and $D_{\varepsilon }$ is the thickness of cell membrane which is sufficiently small. In addition, $P_m$ and $A_m$ denote the concentrations of pPAR and aPAR proteins in the membrane, respectively, while $P_c$ and $A_c$ denote the concentrations of pPAR and aPAR proteins in the cytosol, respectively. $\Delta$ stands for the Laplace operator defined in the bulk domain $\Omega '$ and the cell membrane domain $\Gamma _{\varepsilon }$. We do not consider the flux between cell membrane and extracellular space, so that zero flux boundary condition is assumed in the outer surface of cell membrane.

As $|D_{\varepsilon }|\rightarrow 0$, the model (1.1) can be directly reduced to a bulk-surface model (Fig. 1b(b2)) described as follows:

1.2 $$\begin{aligned} \begin{aligned}&\partial _t{P_m}=D_m\Delta _{\Gamma }{P_m} -F_{\text {off}}(A_m){P_m}+\gamma {P_c}\quad \mathrm {on} \quad \Gamma , \\&\partial _t{P_c}=D_c\Delta {P_c}\quad \mathrm {in}\quad \Omega ', \\&D_c\partial _\nu {P_c}=F_{\text {off}}(A_m){P_m}-\gamma {P_c}\quad \mathrm {on}\quad \Gamma , \\&\partial _t{A_m}=\overline{D}_m\Delta _{\Gamma }{A_m} -\overline{F}_{\text {off}}(P_m){A_m}+{\overline{\gamma }}{A_c}\quad \mathrm {on} \quad \Gamma , \\&\partial _t{A_c}=\overline{D}_c\Delta {A_c}\quad \mathrm {in}\quad \Omega ', \\&D_c\partial _\nu {A_c}=\overline{F}_{\text {off}}(P_m){A_m}-{\overline{\gamma }}{A_c}\quad \mathrm {on}\quad \Gamma , \end{aligned} \end{aligned}$$

where $\Delta _\Gamma$ is the Laplace-Beltrami operator on $\Gamma$.

The kinetic terms, $F_{\text {off}}$ and $\overline{F}_{\text {off}}$, imply the translocation of pPAR by aPAR and aPAR by pPAR from the membrane to the cytosol. In many polarity models, the off-rate functions have been expressed in terms of nonlinear functions, allowing a bi-stable property. In this study, we consider two types of off-rate functions, which have been explored as a Hill function type in Seirin-Lee and Shibata (2015) using

1.3 $$\begin{aligned} F_{\text {off}}(A_m)=\alpha +\frac{K_1{A_m}^2}{K+{A_m}^2}, \qquad \overline{F}_{\text {off}}(P_m)={\overline{\alpha }}+\frac{\overline{K_1}{P_m}^2}{\overline{K}+{P_m}^2}, \end{aligned}$$

and have been studied in Goehring et al. (2011b) through

1.4 $$\begin{aligned} F_{\text {off}}(A_m)=\alpha + K_2{A_m}^2, \qquad \overline{F}_{\text {off}}(P_m)={\overline{\alpha }}+{\widetilde{K}_2}{P_m}^2, \end{aligned}$$

where $\alpha$ and ${\overline{\alpha }}$ are the basal off-rates of pPAR and aPAR, respectively, and $K, K_1$, ${\overline{K}}$, $\overline{K}_1,$ $K_2$, and ${\widetilde{K}}_2$ are positive constants determining the off-rate effects. In addition, $\gamma$ and ${\overline{\gamma }}$ are on-rates parameters. Note that the original models for PAR polarity include advection terms (flow effects) because an acto-myosin contraction causes an advective transport (flow) in both the membrane and cytosol after the symmetry breaking (Goehring et al. 2011b; Niwayama et al. 2011). However, we neglect the advection terms in our model because the flow effect is ceased in the maintenance phase (approximately, 13 minutes later after symmetry breaking) where the polarity domains stop spreading (Munro and Nance 2004; Goehring et al. 2011b). In addition, the purpose of this study is to focus on the long time behavior of the polarity solutions.

In this paper, we explore the high-dimensional polarity models, (1.1) and (1.2), and confirm the existence of stable polarity solutions using numerical simulations. We then prove the existence and stability of such solutions with respect to a high-dimensional case through a simplification of the model (1.1), called a cell membrane periphery model, and a model reduction to a shadow system of the bulk-surface model (1.2). In our study, a rigorous proof for the existence of stable polarity solutions in the aPAR and pPAR polarity models within an arbitrary high-dimensional domain is proposed. Based on our analysis, we further explore how the boundary position of the polarity domains is determined. Our results suggest that the existence and dynamics of the PAR polarity during asymmetric cell division can be understood based on a basic mathematical structure, which should be held universally without dependence on a specific choice of parameter values.

We remark that our mathematical analysis is carried out by making use of the energy functional under the assumption of a large difference of diffusion coefficients of the proteins in the membrane and the cytosol. We refer to Mori et al. (2011) for another approach to identify the polarity boundary in a simpler model called the wave pinning model of two-component reaction–diffusion equations. In the wave pinning model, the authors succeeded in obtaining a reasonable approximation of polarity boundaries in one-dimensional domain by using asymptotic analysis with the membrane diffusion coefficient as a small parameter of the membrane diffusion coefficient. In Cusseddu et al. (2019), a similar analysis is performed on the bulk-surface version of the model in Mori et al. (2011).

## Models and simulations

### Model reductions

#### Nonlinearity of off-rate functions

We here show that the model of (1.4) suggested by Goehring et al. (2011b) is close to the model of (1.3) suggested by Seirin-Lee and Shibata (2015). By using an approximation through a Taylor expansion around small concentration of $A_m$ and small concentration of $P_m$ for the off-rate functions (1.3), respectively, we have

2.1 $$\begin{aligned} \begin{aligned}&F_{\text {off}}(A_m)=\alpha +\frac{K_1{A_m}^2}{K+{A_m}^2}\approx \alpha +\frac{K_1}{K}A_m^2+O(A_m^3), \quad \text { and}\\&\overline{F}_{\text {off}}(P_m)={\overline{\alpha }}+\frac{\overline{K_1}{P_m}^2}{\overline{K}+{P_m}^2}\approx {\overline{\alpha }}+\frac{\overline{K_1}}{\overline{K}}{P_m}^2+O(P_m^3). \end{aligned} \end{aligned}$$

This approximation implies that the model of (1.4) can be considered as a special case of the model of (1.3) by taking $K_2=K_1/K$ and ${\widetilde{K}}_2={\overline{K}}_1/{\overline{K}}$. Furthermore, the two models show little differences in the qualitative dynamics in the long time behavior and it may be sufficient to choose either of them in order to understand the mathematical structure of a stable polarity pattern (see Sect.  2.2). Therefore, we choose the nonlinear off-rate functions by (2.1) when we perform mathematical investigation.

#### A cell membrane periphery model

The diffusion coefficient of protein in the cytosol is generally much larger than that in the membrane (Goehring et al. 2011b; Kuhn et al. 2011). We thus assume that the fast diffusions of aPAR and pPAR in the cytosol lead to a well-mixed state and that the concentrations of aPAR and pPAR in the cytosol quickly approach to a uniform state in the region far from the membrane. Then, as for the polarity patterns in the model systems (1.1) with (1.3) or (1.4), it is reasonable to consider the peripheral region of the membrane (Fig. 1b(b4)). Thus, the model (1.1) can be directly considered in a cell membrane periphery domain by defining the cytoplasmic domain with $\Omega '_{\varepsilon }\equiv \Gamma \times D_{\varepsilon }^{'}$ instead of $\Omega '$, where $D_{\varepsilon }^{'}$ is the sufficiently small cross-sectional area of cytosol region in the periphery of the cell membrane. Note that with the assumption of $|D_{\varepsilon }|=|D_{\varepsilon }^{'}|\ll 1$, we may consider the model (1.1) on a domain where the cell membrane and cytoplasm are overlapped. That is,

2.2 $$\begin{aligned} \begin{aligned}&\partial _t{P_m}=D_m{\Delta }{P_m} -F_{\text {off}}(A_m){P_m}+\gamma {P_c},\\&\partial _t{P_c}=D_c{\Delta }{P_c}+F_{\text {off}}(A_m){P_m}-\gamma {P_c}, \\&\partial _t{A_m}=\overline{D}_m{\Delta }{A_m} -\overline{F}_{\text {off}}(P_m){A_m}+{\overline{\gamma }}{A_c}, \\&\partial _t{A_c}=\overline{D}_c{\Delta }{A_c}+\overline{F}_{\text {off}}(P_m){A_m}-{\overline{\gamma }}{A_c} \end{aligned} \end{aligned}$$

on $\Omega _{\varepsilon }^{'}=\Gamma \times D (\subset \mathbb {R}^{N})$ where $|D|=|D_{\varepsilon }|=|D_{\varepsilon }^{'}|$. Therefore, we can simplify the model (1.1) to the above model defined on a high-dimensional space under the Neumann boundary conditions or periodic boundary conditions.

With setting

$$\begin{aligned} k:=K_1/K ~~(\text {or}~ K_2),\qquad \tau :=(\overline{K}/\overline{K}_1)k ~~(\text {or}~ {\widetilde{K}}_2 k), \end{aligned}$$

in (2.1), and in terms of the new variables and parameters defined by

$$\begin{aligned}&P_m=u_1, ~~P_c=v_1, ~~A_m=u_2,~~A_c=v_2,~~D_m=d_1,~~D_c=D_1, \\&\tau \overline{D}_m=d_2,~~\tau \overline{D}_c=D_2,~~ \gamma =\gamma _1, ~~\tau \overline{\gamma }=\gamma _2,~~\alpha =\alpha _1,~~\tau {\overline{\alpha }}=\alpha _2, \end{aligned}$$

we rewrite the system (2.2) as follows:

2.3 $$\begin{aligned}&\partial _t{u_1}=d_1\Delta {u_1}-(\alpha _1+k u_2^2){u_1}+\gamma _1v_1, \end{aligned}$$

2.4 $$\begin{aligned}&\partial _t{v_1}=D_1\Delta {v_1}+(\alpha _1+k u_2^2){u_1}-\gamma _1v_1, \end{aligned}$$

2.5 $$\begin{aligned}&\tau \partial _t{u_2}={d}_2\Delta {u_2}-(\alpha _2+k u_1^2){u_2}+\gamma _2 {v_2}, \end{aligned}$$

2.6 $$\begin{aligned}&\tau \partial _t{v_2}={D}_2\Delta {v_2}+(\alpha _2+k u_1^2){u_2}-\gamma _2{v_2}, \end{aligned}$$

where

2.7 $$\begin{aligned} d_1<D_1,\qquad d_2<D_2 \end{aligned}$$

in a bounded domain $\Omega (\subset \mathbb {R}^N)$ with the Neumann boundary condition or periodic boundary conditions. The condition (2.7) is due to the fact that the diffusion coefficient of protein in the cytosol is generally larger than that in the membrane (Goehring et al. 2011a, b; Kuhn et al. 2011). $\Omega$ is given to $\Gamma \times D= (0, L)\times D$ where *D* is a bounded domain of $\mathbb {R}^{N-1}$. Figure 1b(b4)-(b6) and 1C show the cases of $N=1, 2, 3$. *L* represents the perimeter length of the cell and |*D*| represents the thickness or cross-sectional area (which does not have to be a disk) of the cell membrane or cytoplasmic domain in two and three dimensional spaces, respectively. We call the system (2.3)–(2.6) *a cell membrane periphery model*. The dynamics of PAR polarity in a *C. elegans* embryo can be understood as the case of a cell membrane periphery model with periodic boundary conditions.

#### A shadow system of bulk-surface model

We introduce a shadow system of the bulk-surface model (1.2) in this subsection. As seen in the later Sect. 3.2, we reduce the stationary equations of the cell periphery model (2.3)–(2.6) to a simpler system. Then, we show that the reduced equations can be obtained as the Euler–Lagrange equations of some energy functional and, interestingly, the shadow system of the bulk-surface model (1.2) with (1.4) has a similar form to the gradient flow of the functional. Thus, the mathematical results for the cell membrane periphery model (2.3)–(2.6) can be directly applied to the shadow system of the bulk-surface model (1.2) with (1.4) (Fig. 1b(b3), (b7))(See Section 3.4).

Let us return to a bulk-surface diffusion model (1.2). Namely, we consider the equation for $P_c$ and $A_c$ in a bulk domain $\Omega '$, where the mass transport takes place on the boundary $\Gamma =\partial \Omega '$. The bulk-surface diffusion with mass transport on the boundary in a non-dimensional form is given by

$$\begin{aligned}&\partial _t{P_m}=D_m\Delta _{\Gamma }{P_m} -F_{\text {off}}(A_m){P_m}+{\gamma P_c}\quad \mathrm {on} \quad \Gamma , \\&\partial _t{P_c}=D_c\Delta {P_c}\quad \mathrm {in}\quad \Omega ', \\&D_c\partial _\nu {P_c}=F_{\text {off}}(A_m){P_m}-{\gamma P_c}\quad \mathrm {on}\quad \Gamma , \\&\partial _t{A_m}=\overline{D}_m\Delta _{\Gamma }{A_m} -{\overline{F}}_{\text {off}}(P_m){A_m}+{{\overline{\gamma }} A_c}\quad \mathrm {on} \quad \Gamma , \\&\partial _t{A_c}=\overline{D}_c\Delta {A_c}\quad \mathrm {in}\quad \Omega ', \\&D_c\partial _\nu {A_c}=\overline{F}_{\text {off}}(P_m){A_m}-{{\overline{\gamma }} A_c}\quad \mathrm {on}\quad \Gamma , \end{aligned}$$

with initial data

$$\begin{aligned} {P_m}(x,0)=p_m(x)\quad (x\in \Gamma ), \quad {P_c}(x,0)=p_c(x)\quad (x\in \Omega '), \\ {A_m}(x,0)=a_m(x)\quad (x\in \Gamma ), \quad {A_c}(x,0)=a_c(x)\quad (x\in \Omega '). \end{aligned}$$

This system has the following conservation of mass:

$$\begin{aligned}&\int _{\Omega '}{P_c}(x,t)dx+\int _{\Gamma }{P_m}(x,t)dx_{\Gamma }= \int _{\Omega '}{p_c}(x)dx+\int _{\Gamma }{p_m}(x)dx_{\Gamma }, \\&\int _{\Omega '}{A_c}(x,t)dx+\int _{\Gamma }{A_m}(x,t)dx_{\Gamma }= \int _{\Omega '}{a_c}(x)dx+\int _{\Gamma }{a_m}(x)dx_{\Gamma }, \end{aligned}$$

because

$$\begin{aligned} \frac{d}{dt}\left( \int _{\Omega '}{P_c}dx+\int _{\Gamma }{P_m}dx_{\Gamma }\right) =0, \qquad \frac{d}{dt}\left( \int _{\Omega '}{A_c}dx+\int _{\Gamma }{A_m}dx_{\Gamma }\right) =0. \end{aligned}$$

We set

$$\begin{aligned}&{{\widetilde{m}}}_1:=\frac{1}{|\Gamma |}\left( \int _{\Omega '}{P_c}dx+\int _{\Gamma }{P_m}dx{ _\Gamma }\right) ,\\&{ \widetilde{m}}_2:=\frac{1}{|\Gamma |}\left( \int _{\Omega '}{A_c}dx+\int _{\Gamma }{A_m}dx{ _\Gamma }\right) . \end{aligned}$$

We reduce the equations to a shadow system on $\Gamma$ by taking $D_c, \overline{D}_c\rightarrow \infty$ (Fig. 1b(b2),(b3)) as

$$\begin{aligned}&{P_c}(x,t)\rightarrow \xi (t):=\frac{1}{|\Omega '|}\int _{\Omega '} {P_c}(x,t)dx, \\&{A_c}(x,t)\rightarrow \eta (t):=\frac{1}{|\Omega '|}\int _{\Omega '} {A_c}(x,t)dx, \end{aligned}$$

and the system turns to be

2.8 $$\begin{aligned} \begin{aligned}&\partial _t{P_m}=D_m\Delta _{\Gamma }{P_m} - F_{\text {off}}(A_m){P_m} +{\gamma \xi } \quad \mathrm {on} \quad \Gamma , \\&\partial _t{A_m}=\overline{D}_m\Delta _{\Gamma }{A_m} - {\overline{F}}_{\text {off}}(P_m){A_m}+{{\overline{\gamma }} \eta } \quad \mathrm {on} \quad \Gamma , \end{aligned} \end{aligned}$$

and

2.9 $$\begin{aligned} \begin{aligned}&{ {\widetilde{m}}}_1=\frac{|\Omega '|}{|\Gamma |}\xi +\frac{1}{|\Gamma |}\int _{\Gamma }{P_m}(x,t)dx,\\&{ {\widetilde{m}}}_2=\frac{|\Omega '|}{|\Gamma |}{ \eta }+\frac{1}{|\Gamma |}\int _{\Gamma }{A_m}(x,t)dx. \end{aligned} \end{aligned}$$

Then we have equations for $\xi$ and $\eta$ as

$$\begin{aligned} \dot{\xi }&=\frac{1}{|\Omega '|} \int _{\Gamma } F_{off}(A_m)P_{m}dx+\gamma \frac{|\Gamma |}{|\Omega '|} \xi , \\ \dot{\eta }&=\frac{1}{|\Omega '|} \int _{\Gamma } \overline{F}_{off}(P_m)A_{m}dx+{\overline{\gamma }} \frac{|\Gamma |}{|\Omega '|} \eta , \end{aligned}$$

which are derived by taking the spatial average of the bulk equations for $P_c$ and $A_c$ and applying the Green formula. However, these equations for $\xi$ and $\eta$ are obtained by differentiating (2.9) with respect to *t* and using (2.8). Hence, it suffices to handle (2.8) under the constraint (2.9) as the shadow system.

Introducing the new variables for the model (2.8) with the off-rate function (1.3), we reduce the parameters as

$$\begin{aligned}&u={P_m}/\overline{K}^{1/2}, \qquad v={P_m}/{K}^{1/2}, \\&\xi _1=\frac{|\Omega '|}{|\Gamma |}\frac{\xi }{\overline{K}^{1/2}}, \qquad \xi _2=\frac{|\Omega '|}{|\Gamma |}\frac{\eta }{{K}^{1/2}}, \qquad t'=t/\overline{K}_1, \end{aligned}$$

and let us set

2.10 $$\begin{aligned} \begin{aligned}&d_1=D_m\overline{K}_1, \quad d_2=\overline{D}_m{K}_1, \quad k=K_1\overline{K}_1, \quad \tau =K_1/\overline{K}_1\\&\alpha _1=\alpha \overline{K}_1,\quad \alpha _2=\overline{\alpha }{K}_1, \quad \gamma _1=\gamma \overline{K}_1 \frac{|\Gamma |}{|\Omega '|},\quad \gamma _2=\overline{\gamma }{K}_1, \frac{|\Gamma |}{|\Omega '|},\\&{ {\widetilde{M}}}_1={ {\widetilde{m}}}_1/\overline{K}^{1/2}, \quad { {\widetilde{M}}}_2={ {\widetilde{m}}}_2/{K}^{1/2}. \end{aligned} \end{aligned}$$

Dropping $'$ of $t'$ in the equations for *u* and *v* leads to

2.11 $$\begin{aligned}&\partial _t u=d_1\Delta _\Gamma u-\left( \alpha _1+\frac{k v^2}{1+v^2}\right) u+\gamma _1\xi _1, \end{aligned}$$

2.12 $$\begin{aligned}&\tau \partial _t v=d_2\Delta _\Gamma v-\left( \alpha _2+\frac{k u^2}{1+u^2}\right) v+\gamma _2\xi _2, \end{aligned}$$

with

2.13 $$\begin{aligned} { {\widetilde{M}}}_1=\xi _1+\frac{1}{|\Gamma |}\int _{\Gamma }u dx_\Gamma \qquad { \widetilde{M}}_2=\xi _2+\frac{1}{|\Gamma |}\int _{\Gamma }v dx_\Gamma . \end{aligned}$$

In a similar manner to the above derivation, we can also derive the shadow system of the cell membrane periphery model (2.3)–(2.6) in a bounded domain $\Omega \subset \mathbb {R}^N$ as

2.14 $$\begin{aligned}&\partial _t u=d_1\Delta u-\left( \alpha _1+kv^2\right) u+\gamma _1\xi _1 ~~\mathrm {in}~~\Omega , \end{aligned}$$

2.15 $$\begin{aligned}&\tau \partial _t v=d_2\Delta v-\left( \alpha _2+ku^2\right) v+\gamma _2\xi _2 ~~\mathrm {in}~~\Omega , \end{aligned}$$

with

2.16 $$\begin{aligned} { M_1:}=\xi _1+\frac{1}{|\Omega |}\int _{\Omega }u dx, \qquad { M_2:}=\xi _2+\frac{1}{|\Omega |}\int _{\Omega }v dx. \end{aligned}$$

In the sequel, we are concerned with the shadow system of (2.3)–(2.6) in $\Omega$,

2.17 $$\begin{aligned}&\partial _t u=d_1\Delta u-\left( \alpha _1+kv^2\right) u+\gamma _1({ M_1}-\langle u\rangle ) ~~\mathrm {in}~~\Omega , \end{aligned}$$

2.18 $$\begin{aligned}&\tau \partial _t v=d_2\Delta v-\left( \alpha _2+ku^2\right) v+\gamma _2({ M_2}-\langle v\rangle ) ~~\mathrm {in}~~\Omega , \end{aligned}$$

with

2.19 $$\begin{aligned} \frac{\partial u}{\partial \varvec{n}}=\frac{\partial v}{\partial \varvec{n}}=0 \quad \mathrm {on} \quad \partial \Omega , \end{aligned}$$

where

$$\begin{aligned} \langle \cdot \rangle :=\frac{1}{|\Omega |}\int _\Omega \cdot ~dx. \end{aligned}$$

Note that the approximation of shadow system induces a model defined on a surface domain which does not include the thickness of cell membrane. However, we can deal with the shadow system mathematically similarly to a cell membrane periphery model assumed sufficiently small *D* as shown in Fig. 1b. As the simplest case, we can handle the above system in $\Omega =(0,L)$ with periodic boundary condition. Although (2.17)–(2.18) with (2.19) are simplified model equations, it is very useful to perform numerical simulations with low numerical costs in higher-dimensional domains. We will numerically show that the solution of this shadow system exhibits similar behaviors as the other models in the following section.

**Fig. 2:**
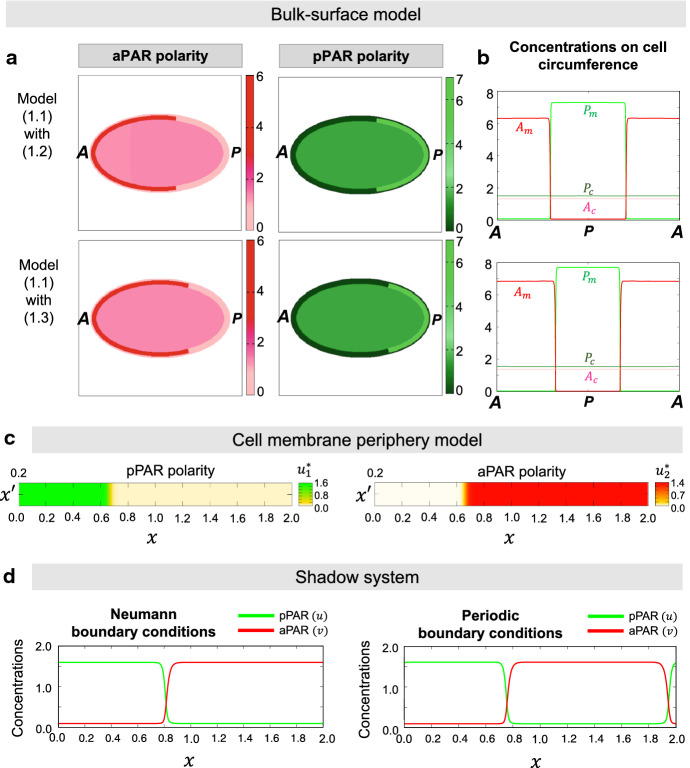
Numerical simulations and polarity solutions. **a** Numerical results for bulk-surface model (1.2) are shown. The color maps indicate the concentrations of aPAR and pPAR on the cytosol and membrane in each region. The detailed parameter values are as follows: $D_m=7.2\times 10^{-7} ,~\overline{D}_m=1.652 \times 10^{-6} , ~D_c=\overline{D}_c= 3.6\times 10^{-4}, ~\gamma ={\overline{\gamma }}= 0.3, ~\alpha ={\overline{\alpha }}=0.06 , ~K_1=\overline{K}_1=0.4, ~K=\overline{K}=0.05$ for model (1.2) with (1.3), and $\gamma _1=\gamma _2=0.3, ~\alpha _1=\alpha _2=0.06, ~\kappa =0.4$ for model (1.2) with (1.4). **b** The concentrations of aPAR and pPAR on the cell circumference calculated in (**a**) are plotted. **c** Stable nonconstant equilibrium solutions of the cell membrane periphery model (2.3)–(2.6) on $\Omega =L\times D$, where $L=(0, 2)$ and $D=[0, 0.2]$. The detailed parameter values are given as $d_1=7.2\times 10^{-6}, ~d_2=1.652 \times 10^{-5}, ~D_1=D_2= 3.6\times 10^{-3}, ~\gamma _1=2.6, ~\gamma _2=2.0, ~\alpha _1=\alpha _2=0.06, ~\kappa =0.4, ~\tau =1.0$, $m_1=0.737115$, and $m_2=1.0293325$. (D) Stable nonconstant equilibrium solutions of the shadow system (2.17)–(2.18) on $\Omega =[0, 2]$ under the Neumann boundary conditions and the periodic boundary conditions. The detailed parameters are given to $d_1=7.2 \times 10^{-6}, d_2=1.652 \times 10^{-5}$, $\tau =1.0$, $\alpha _1=\alpha _2=0.6$, $\gamma _1=2.6, \gamma _2=2.0$, $M_1=0.737115$, and $M_2=1.0293325$ (color figure online)

###  Numerical simulation results

In this study, we consider the long time behavior of segregation pattern of $P_m$ and $A_m$ in the system, namely, a stable steady-state polarity patterns of pPAR and aPAR. Thus, we first compare the quantitative dynamics of the two bulk-surface models (1.2) with (1.3) and (1.4) for the long time behavior (see Appendix A for the numerical method and scheme for solving bulk-surface model). Figure 2a shows that both of models successfully generate exclusive polarity patterns and that there are no notable differences between the two models. Furthermore, as shown in Fig. 2b of the profile of the membrane PAR proteins, the two polarity domains are overlapped with a small interface region. This may allow us to assume that the off-rate effect of $P_m$ by $A_m$ is negligible in the region where the concentration of $P_m$ is low (i.e. aPAR dominated region) while the off-rate effect is likely to be dominated in the region where the concentration of $P_m$ is high (i.e. pPAR dominated region). Similarly, the off-rate effect of $A_m$ by $P_m$ is likely to be dominated in the region where the concentration of $A_m$ is high (i.e. aPAR dominated region). The approximation by a Taylor expansion in (2.1) may imply this phenomenon.

Figure 2c shows stable polarity solutions of the cell membrane periphery model (2.3)–(2.6) on $\Omega (\subset \mathbb {R}^2)$ under the Neumann boundary conditions. Fig. 2d shows nonconstant equilibrium solutions of shadow system (2.17)–(2.18) on $\Omega (\subset \mathbb {R}^1)$ for both Neumann and periodic boundary conditions. The simulation results show, at least apparently, that there are no differences in the qualitative dynamics of polarity solutions between the bulk-surface model, the cell membrane periphery model, and the shadow system.

Note that the kinetic parameter values used in the representative simulations were chosen as arbitrary values which basically satisfy the condition that two stable equilibria exist in kinetic equations. The diffusion coefficients were chosen as the values of biologically feasible scale based on the data of *C. elegans* embryo (Goehring et al. 2011a; Kuhn et al. 2011; Seirin-Lee 2021). Although we showed the polarity solutions with representative parameter values, our rigorous analysis and mathematical results of this paper support that the existence of polarity pattern is less sensitive to parameter choice and holds over a wide range of the parameter space.

## Long time behavior of polarity solutions

### Basic properties of solutions to the system

Let $\Omega$ be a bounded domain in $\mathbb {R}^N$ with smooth boundary $\partial \Omega$. In this section, we consider the system (2.3)–(2.6) in $\Omega$ with the Neumann boundary conditions

3.1 $$\begin{aligned} \frac{\partial u_1}{\partial \varvec{n}}=\frac{\partial u_2}{\partial \varvec{n}}= \frac{\partial v_1}{\partial \varvec{n}}=\frac{\partial v_2}{\partial \varvec{n}}=0 \quad (x\in \partial \Omega ). \end{aligned}$$

Assume nonnegative continuous initial data

3.2 $$\begin{aligned} {\left\{ \begin{array}{ll} u_1(x,0)=u_{1,0}(x)\ge 0, \quad u_2(x,0)=u_{2,0}(x)\ge 0, \quad \\ v_1(x,0)=v_{1,0}(x)\ge 0, \quad v_2(x,0)=v_{2,0}(x)\ge 0 \end{array}\right. } \quad (x\in \overline{\Omega }), \end{aligned}$$

where $u_{i,0}, v_{i,0}~(i=1,2)$ are not identically zero. We aim to show the fundamental mathematical results on the positivity and global boundedness of the solution to the cell periphery model (2.3)–(2.6) with (3.1) and (3.2). The results here is easily interpreted to the case of periodic boundary condition.

The first lemma assures the positivity of the solution.

#### Lemma 3.1

The system (2.3)–(2.6) in $\Omega$ with (3.1) and (3.2) has a unique classical solution $(u_1(x,t), u_2(x,t), v_1(x,t), v_2(x,t))$ satisfying

$$\begin{aligned} u_1(x,t), u_2(x,t), v_1(x,t), v_2(x,t)>0~(x\in \overline{\Omega }) \end{aligned}$$

for $t>0$ in the maximal interval.

Next, in order to prove that the solution can be extended globally in time, we introduce the new variables $z_1=(d_1/D_1)u_1+v_1$ and $z_2=(d_2/D_2)u_2+v_2$ and convert the system (2.3)–(2.6) to

3.3 $$\begin{aligned}&\partial _t{u_1}=d_1\Delta {u_1}-(\alpha _1+k u_2^2){u_1}-(\gamma _1 d_1/D_1)u_1+\gamma _1z_1, \end{aligned}$$

3.4 $$\begin{aligned}&(1-d_1/D_1)\partial _t{u_1}+\partial _t z_1=D_1\Delta z_1, \end{aligned}$$

3.5 $$\begin{aligned}&\tau \partial _t{u_2}={d}_2\Delta {u_2}-(\alpha _2+k u_1^2){u_2}-(\gamma _2 d_2/D_2)u_2+\gamma _2z_2, \end{aligned}$$

3.6 $$\begin{aligned}&\tau (1-d_2/D_2)\partial _t{u_2}+\tau \partial _t z_2=D_2\Delta z_2. \end{aligned}$$

Define

3.7 $$\begin{aligned} {{\mathcal {E}}}(\varvec{u}, \varvec{z}):=&\int _{\Omega } \left[ \frac{d_1}{2}|\nabla u_1|^2+\frac{d_2}{2}|\nabla u_2|^2 +\frac{\alpha _1+\gamma _1d_1/D_1}{2}u_1^2 \right. \nonumber \\&\qquad \left. +\frac{\alpha _2+\gamma _2d_2/D_2}{2}{u_2^2} +\frac{k}{2}u_1^2u_2^2+\frac{\theta _1}{2}z_1^2+\frac{\theta _2}{2}z_2^2\right] dx, \end{aligned}$$

where $\varvec{u}=(u_1, u_2), \varvec{z}=(z_1,z_2)$, and $\theta$: $(i = 1, 2)$ are given in (3.9). Then, we can prove that ${{\mathcal {E}}}$ plays as a Lyapunov function of (3.3)–(3.6) and obtain the next result.

#### Lemma 3.2

Given positive parameters $d_i, D_i, \alpha _i, \gamma _i~(i=1,2)$ and $\tau$ with $d_i<D_i~(i=1,2)$, there exists a constant $C_1$, depending on the initial data, such that

3.8 $$\begin{aligned}&\Vert u_1(\cdot ,t)\Vert _{H^1}^2+\Vert u_2(\cdot ,t)\Vert _{H^1}^2+\Vert z_1(\cdot ,t)\Vert _{L^2}^2+\Vert z_2(\cdot ,t)\Vert _{L^2}^2 \nonumber \\&\qquad +\int _{0}^t \{\Vert \partial _t u_1(\cdot ,t')\Vert _{L^2}^2+\tau \Vert \partial _t u_2(\cdot ,t') \Vert ^2 \nonumber \\&\qquad \qquad +\theta _1D_1\Vert \nabla (z_1(\cdot , t')\Vert _{L^2}^2+(\theta _2/\tau )D_2\Vert \nabla z_2(\cdot , t')\Vert _{L^2}^2\}dt' \le C_1 \end{aligned}$$

where

3.9 $$\begin{aligned} \theta _1:=\gamma _1/(1-d_1/D_1),\qquad \theta _2:=\gamma _2/(1-d_2/D_2). \end{aligned}$$

Using this lemma, we get to the global boundedness of the solution;

#### Lemma 3.3

Let $\Omega \subset \mathbb {R}^N$, $1\le N\le 3$. Then the solution $(u_1(\cdot ,t), v_1(\cdot ,t), u_2(\cdot ,t), v_2(\cdot ,t))$ is uniformly bounded in *t*, that is, there is a constant $C_m>0$ such that

$$\begin{aligned} \Vert u_1(\cdot ,t)\Vert _{L^\infty }, ~\Vert v_1(\cdot ,t)\Vert _{L^\infty }, ~\Vert u_2(\cdot ,t)\Vert _{L^\infty },~\Vert v_2(\cdot ,t)\Vert _{L^\infty }\le C_m\quad (t\ge 0). \end{aligned}$$

The proofs of the above three lemmas are given in Appendix B.1.

#### Remark 3.1

In the work of Latos et al. (2018), a similar argument for the proof of the uniform boundedness can be found. They study a two-component system having the nonlinear terms with linear growth at infinity by which the uniform boundedness can be shown without the restriction of space dimensions. Another approach is also found in Jimbo and Morita (2013) for the similar system to Latos et al. (2018).

### Stability of equilibrium solutions

From the system (2.3)–(2.6) with (3.1), we see

$$\begin{aligned} \frac{d}{dt}\int _{\Omega }(u_1(x,t)+v_1(x,t))dx=0,\qquad \frac{d}{dt}\int _{\Omega }(u_2(x,t)+v_2(x,t))dx=0, \end{aligned}$$

so that the system allows mass conservations

$$\begin{aligned} \int _{\Omega }(u_1(x,t)+v_1(x,t))dx=\mathrm {constant},\qquad \int _{\Omega }(u_2(x,t)+v_2(x,t))dx=\mathrm {constant}. \end{aligned}$$

We set

$$\begin{aligned} m_1:=\langle u_1 \rangle +\langle v_1 \rangle , \qquad m_2:= \langle u_2\rangle +\langle v_2\rangle , \end{aligned}$$

where $\langle \cdot \rangle :=(1/|\Omega |)\int _{\Omega } \cdot ~dx$. This mass conservation is expressed in the system (3.3)–(3.6) as

$$\begin{aligned} m_1=(1-d_1/D_1)\langle u_1\rangle +\langle z_1\rangle , \qquad m_2=(1-d_2/D_2) \langle u_2\rangle +\langle z_2\rangle . \end{aligned}$$

In this subsection, we investigate the stationary problem of the system (2.3)–(2.6), that is, of the system (3.3)–(3.6),

$$\begin{aligned}&d_1\Delta {u_1}-(\alpha _1+k u_2^2){u_1}-(\gamma _1 d_1/D_1)u_1+\gamma _1z_1=0, \qquad \Delta z_1=0, \\&{d}_2\Delta {u_2}-(\alpha _2+k u_1^2){u_2}-(\gamma _2 d_2/D_2)u_2+\gamma _2 z_2=0, \qquad \Delta z_2=0, \end{aligned}$$

with

$$\begin{aligned} m_i=(1-d_i/D_i)\langle u_i\rangle +\langle z_i\rangle \qquad (i=1,2). \end{aligned}$$

These equations turn out to be

3.10 $$\begin{aligned} \begin{aligned}&d_1\Delta {u_1}-(\alpha _1+k u_2^2){u_1}-(\gamma _1 d_1/D_1)u_1+\gamma _1 \{m_1-(1-d_1/D_1)\langle u_1\rangle \}=0, \\&{d}_2\Delta {u_2}-(\alpha _2+k u_1^2){u_2}-(\gamma _2 d_2/D_2)u_2+\gamma _2 \{m_2-(1-d_2/D_2)\langle u_2\rangle \}=0. \end{aligned}\nonumber \\ \end{aligned}$$

We put

3.11 $$\begin{aligned} \begin{aligned} \beta _i:=\alpha _i+\gamma _id_i/D_i,\quad M_i:=\frac{m_i}{1-d_i/D_i}\qquad (i=1,2), \end{aligned} \end{aligned}$$

and let $\theta _i~(i=1,2)$ be same as in Lemma 3.2.

The system (3.10) has a variational structure. As a matter of fact, consider the following functional of $\varvec{u}=(u_1,u_2)$.

3.12 $$\begin{aligned} {{\mathcal {E}}}_s(\varvec{u}) :=&\int _{\Omega }\left\{ \frac{d_1}{2}|\nabla u_1|^2+\frac{d_2}{2}|\nabla u_2|^2+ \frac{\beta _1}{2}u_1^2+\frac{\beta _2}{2}u_2^2+\frac{k}{2} u_1^2u_2^2\right\} dx \nonumber \\&+\frac{\gamma _1(1-d_1/D_1)}{2}|\Omega |\left( M_1-\langle u_1\rangle \right) ^2+ \frac{\gamma _2(1-d_2/D_2)}{2}|\Omega |\left( M_2-\langle u_2\rangle \right) ^2. \end{aligned}$$

Then, it is easy to see that (3.10) with the Neumann boundary conditions is the Euler-Lagrange equation of ${{\mathcal {E}}}_s$. Moreover, the following time evolution system

3.13 $$\begin{aligned} \begin{aligned}&\partial _tu_1=d_1\Delta {u_1}-(\alpha _1+k u_2^2){u_1}-(\gamma _1 d_1/D_1)u_1+\gamma _1\{m_1-(1-d_1/D_1)\langle u_1\rangle \}, \\&\partial _t u_2={d}_2\Delta {u_2}-(\alpha _2+k u_1^2){u_2}-(\gamma _2 d_2/D_2)u_2+\gamma _2 \{m_2-(1-d_2/D_2)\langle u_2\rangle \} \end{aligned}\nonumber \\ \end{aligned}$$

serves as the gradient flow of the energy functional ${{\mathcal {E}}}_s$.

We note that corresponding to a solution $(u_1^*, u_2^*)$ of (3.10),

$$\begin{aligned} (u_1^*, z_1^*, u_2^*, z_2^*)=(u_1^*, ~m_1-(1-d_1/D_1)\langle u_1^* \rangle , ~u_2^*, ~m_2-(1-d_2/D_2)\langle u_2^* \rangle ) \end{aligned}$$

provides an equilibrium solution to (3.3)–(3.6). In the rest of this subsection, we show that the stability of $(u_1^*, z_1^*, u_2^*, z_2^*)$ is closely related to that of $(u_1^*, u_2^*)$ in (3.10).

We first observe some property of a local minimizer of ${{\mathcal {E}}}_s$.

#### Lemma 3.4

Let $\varvec{u}^*=(u^*_1, u^*_2)$ be a local minimizer of ${{\mathcal {E}}}_s(\varvec{u})~(\varvec{u}\in H^1(\Omega )^2)$. Then, there exists an $\varepsilon _1>0$ such that for any $\varepsilon \in (0,\varepsilon _1/4]$, we can take $\delta _1=\delta _1(\varepsilon )>0$ so that $\Vert \varvec{u}-\varvec{u}^*\Vert _{H^1}<\varepsilon$ holds if ${{\mathcal {E}}}_s(\varvec{u})-{{\mathcal {E}}}_s(\varvec{u}^*)<\delta _1$ with $\Vert \varvec{u}-\varvec{u}^*\Vert _{H^1}<\varepsilon _1$.

#### Proof

By a slight modification of the proof of Lemma 7 of Latos and Suzuki (2014), we can get to the assertion of the lemma. Thus, we omit the details. $\square$

We decompose

$$\begin{aligned} z_i=\langle z_i\rangle +z_i^Q,\quad z_i^Q:=z_i-\langle z_i\rangle \quad (i=1,2). \end{aligned}$$

Then

$$\begin{aligned} \Vert z_i\Vert _{L^2}^2&=\langle z_i\rangle ^2+\Vert z^Q\Vert _{L^2}^2= (m_i-(1-d_i/D_i)\langle u_i\rangle )^2+\Vert z^Q\Vert _{L^2}^2 \\&= (1-d_i/D_i)^2(M_i-\langle u_i\rangle )^2+\Vert z^Q\Vert _{L^2}^2 \end{aligned}$$

yields

3.14 $$\begin{aligned} {{\mathcal {E}}}(\varvec{u},\varvec{z})={{\mathcal {E}}}_s(\varvec{u})+\frac{\theta _1}{2}\Vert z_1^Q\Vert ^2_{L^2}+\frac{\theta _2}{2}\Vert z_2^Q\Vert _{L^2}^2, \end{aligned}$$

where $\theta _i~(i=1,2)$ are as in (3.9). From the next lemma, we see that the stability of a solution $(\varvec{u}^*, \varvec{z}^*)$ is inherited from that of $\varvec{u}^*$.

#### Lemma 3.5

Let $\varvec{u}^*$ of (3.12) be a local minimizer and let $\varvec{z}^*=(z_1^*, z_2^*)$ be defined as $z_i^*:=m_i-(1-d_i/D_i)\langle u^*_i\rangle \quad (i=1,2).$ Then, given $\varepsilon >0$, there exists $\delta >0$ such that

3.15 $$\begin{aligned} \Vert (\varvec{u}(\cdot ,0), \varvec{z}(\cdot ,0))-(\varvec{u}^*, \varvec{z}^*)\Vert _{H^1}<\delta \end{aligned}$$

implies

$$\begin{aligned} \Vert (\varvec{u}(\cdot ,t), \varvec{z}(\cdot ,t))-(\varvec{u}^*, \varvec{z}^*)\Vert _{H^1}<\widetilde{C}\varepsilon \quad (t\ge 0), \end{aligned}$$

for a constant $$\widetilde{C}>0$$.

The proof of this lemma is given in Appendix B.2.

### Existence of stable nonconstant solutions

**Fig. 3:**
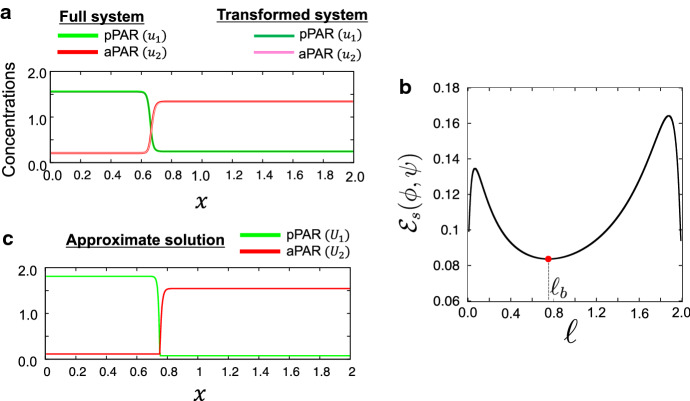
Stable nonconstant equilibrium solutions and minimal energy at the boundary of polarity domains. **a** The solutions to the full system (2.3)–(2.6) shown in Fig. 2c are plotted at $x'=0.1$. With the same parameter values, the stationary solutions of the transformed system (3.13) in a one-dimensional space are plotted. The solutions for both systems mostly overlapped. **b** Energy function (3.24). The red point indicates the location of minimal energy and is at (0.750762, 0.083651). In addition, $\ell _b$ is the location where the energy function (3.24) is at minimum, *i.e.* $\ell _b=0.750762$. **c** Approximate solution given in (3.21) is plotted using $\ell =\ell _b$. The detailed parameter values are the same for **a**–**c** and are given as $d_1=7.2\times 10^{-6}, ~d_2=1.652 \times 10^{-5}, ~D_1=D_2= 3.6\times 10^{-3}, ~\gamma _1=2.6, ~\gamma _2=2.0, ~\alpha _1=\alpha _2=0.06, ~\kappa =0.4, ~\tau =1.0$, $m_1=0.737115$, and $m_2=1.0293325$ (color figure online)

In Sect. 2.2, we confirmed numerically that there exist stable polarity solutions in the cell periphery model (2.3)–(2.6) in Fig. 2c. One can also find that the stable equilibrium solutions in the system (2.3)–(2.6) and the transformed system (3.13) are very consistent, as shown in Fig. 3a. As seen below, the system (3.13) of two variables gives a good mathematical simplification for rigorous analysis, while it is very useful to understand the dynamics of polarity patterns to a stationary state with reducing a numerical cost.

In this subsection, we prove the existence of stable nonconstant equilibrium solutions of (2.3)–(2.6) with (3.1) in a parameter regime. To this end, in view of Lemma 3.4, it suffices to show the existence of a nonconstant minimizer of the energy functional ${{\mathcal {E}}}_s$ of (3.12). As the first step, we ensures the existence of a positive minimizer of ${{\mathcal {E}}}_s$.

#### Lemma 3.6

There is a minimizer $\varvec{u}^*=(u_1^*, u_2^*)$ of ${{\mathcal {E}}}_s$ satisfying $u_i^*(x)>0~(x\in \overline{\Omega }), i=1, 2$.

#### Proof

By the direct method of calculus of variations, we have a minimizer $\varvec{u}^*=(u_1^*, u_2^*)$ of ${{\mathcal {E}}}_s$. We easily exclude the case $u_1^*\le 0, ~u_1^*\not \equiv 0$ or $u_2^*\le 0, u_2^*\not \equiv 0$. Indeed, if $u_1^*\le 0 ~u_1^*\not \equiv 0$, then ${{\mathcal {E}}}_s(-u_1^*, u_2^*)<{{\mathcal {E}}}_s(u_1^*, u_2^*)$, a contradiction. Similarly, $u_2^*\le 0,~u_2^* \not \equiv 0$ cannot happen. This implies that each $u_i^*$ has a positive maximum or $u_i\equiv 0$. Since $(u_1^*, u_2^*)$ satisfies (3.10), $u_i\equiv 0~(i=1,2)$ is excluded. By the Hopf lemma, we see that the maximum point of $u_i^*$ exsits in the interior of the domain unless $u_i^*$ is constant. Moreover,

$$\begin{aligned} (1-d_i/D_i)\langle u_i^*\rangle < m_i, \qquad i=1,2 \end{aligned}$$

hold by applying the maximum principle to the first and second equations of (3.10) (use a contradiction argument for the proof). We exclude the case that $u_i^*$ takes the non-positive minimum. If it happens, the minimum is achieved in the interior of $\Omega$ by the Hopf lemma. Let $x_m\in \Omega$ be such a point. Then

$$\begin{aligned} d_i\Delta u_i-\alpha _i u_i -ku_j^2u_i+\gamma _i(m_i-(1-d_i/D_i)\langle u_i^*\rangle )>0 \end{aligned}$$

at $x=x_m$, which is a contradiction. In conclusion, $u_i^*>0~(i=1,2)$. $\square$

We next consider a positive constant equilibrium solution, which is obtained by a solution of

3.16 $$\begin{aligned} \begin{aligned}&(\alpha _1+\gamma _1d_1/D_1)\xi +k\xi \eta ^2-\gamma _1(m_1-(1-d_1/D_1)\xi )=0,\\&(\alpha _2+\gamma _2d_2/D_2)\eta +k\xi ^2\eta -\gamma _2(m_2-(1-d_2/D_2)\eta )=0. \end{aligned} \end{aligned}$$

Given solution $(\xi ,\eta )$ to the system (3.16), we have

3.17 $$\begin{aligned}&{{\mathcal {E}}}_s(\xi ,\eta )= |\Omega |\left\{ \frac{\beta _1}{2}\xi ^2+\frac{\beta _2}{2}\eta ^2+\frac{k}{2} \xi ^2\eta ^2 \right. \nonumber \\&\left. +\frac{\theta _1}{2}\left( m_1-(1-d_1/D_1)\xi \right) ^2+ \frac{\theta _2}{2}\left( m_2-(1-d_2/D_2)\eta \right) ^2\right\} , \end{aligned}$$

where $\beta _i$ and $\theta _i$ $(i=1,2)$ are defined in (3.11) and (3.9), respectively. We easily see that for fixed $m_i~(i=1,2)$ and *k*, it is impossible to realize the three conditions

$$\begin{aligned} \xi ^2\eta ^2=0, \quad m_1-(1-d_1/D_1)\xi =0, \quad \text{ and }\quad m_2-(1-d_2/D_2)\eta =0, \end{aligned}$$

simultaneously. Hence, if we found a family of nonnegative functions parametrized by, say $\varepsilon >0$, as $\{(u_1^\varepsilon , u_2^\varepsilon )\}_{\varepsilon >0}$ satisfying

$$\begin{aligned} {{\mathcal {E}}}_s(u_1^\varepsilon , u_2^\varepsilon )\rightarrow 0 \quad (\varepsilon \downarrow 0), \end{aligned}$$

${{\mathcal {E}}}_s$ allows a nonconstant minimizer for sufficiently small $\varepsilon$. Moreover, applying Lemma 3.5, we can assert the existence of a stable nonconstant positive equilibrium of the 4-component system of (2.3)–(2.6).

However, we are interested in the profile of the nonconstant minimizer. As a matter of fact, in numerical simulations, the spatial segregation pattern of $u_1$ and $u_2$ can be observed. Considering those simulations, we expect that the interface separating two regions of $u_1$ and $u_2$ emerges in the singular limit by varying appropriate parameters. Here we aim to construct an approximate solution with less energy than any constant solution and identify the location of the interface by minimizing the energy of the approximate solution. Our goal is to provide a reasonable approximation matching with the results of numerics.

Let us set

3.18 $$\begin{aligned} \omega _i:=\sqrt{d_i/\beta _i}, \qquad i=1,2, \end{aligned}$$

and

3.19 $$\begin{aligned} \begin{aligned} \mu _1(\ell ):=&\frac{m_1}{ \frac{1-d_1/D_1}{L}\{\ell - \omega _1\tanh (\ell /\omega _1)\}+\beta _1/\gamma _1}, \\ \mu _2(\ell ):=&\frac{m_2}{ \frac{1-d_2/D_2}{L}\{ L-\ell - \omega _2\tanh ((L-\ell )/\omega _2)\}+\beta _2/\gamma _2}, \end{aligned} \end{aligned}$$

where $\ell$ serves as a location of an interface in the approximate solution. We consider the parameter regime

3.20 $$\begin{aligned} d_i\ll D_i,~~\beta _i \ll 1\quad (i=1,2), \qquad \ell>\omega _1, \quad L-\ell >\omega _2. \end{aligned}$$

Define

3.21 $$\begin{aligned} \begin{aligned}&U_1(x_1;\ell ):=\mu _1(\ell ) \left( 1-\frac{\cosh (x_1/\omega _1)}{\cosh (\ell /\omega _1)}\right) , \\&U_2(x_1;\ell ):=\mu _2(\ell ) \left( 1-\frac{\cosh ((L-x_1)/\omega _2)}{\cosh ((L-\ell )/\omega _2)}\right) , \end{aligned} \end{aligned}$$

and use the following test functions:

3.22 $$\begin{aligned}&\phi (x_1):={\left\{ \begin{array}{ll} U_1(x_1;\ell )+\delta U_1 &{} (0\le x_1\le \ell ), \\ \delta U_1 &{} (\ell \le x_1\le L), \end{array}\right. } \end{aligned}$$

3.23 $$\begin{aligned}&\psi (x_1):={\left\{ \begin{array}{ll} \delta U_2 &{} (0\le x_1\le \ell ), \\ U_2(x_1;\ell )+\delta U_2&{} (\ell \le x_1\le L). \end{array}\right. } \end{aligned}$$

We compute the energy ${{\mathcal {E}}}_s(\phi ,\psi )$. By a lengthy but straightforward computation (for the details, see Appendix C), we obtain

3.24 $$\begin{aligned} \begin{aligned}&{{\mathcal {E}}}_s(\phi ,\psi ) = \frac{|D|\beta _1}{2}\mu _1(\ell )^2 \{\ell (1+\rho _1)^2+(L-\ell )\rho _1^2-(1+2\rho _1)\omega _1\tanh (\ell /\omega _1)\} \\&\quad + \frac{|D|\beta _2}{2}\mu _2(\ell )^2 \{(L-\ell )(1+\rho _2)^2+\ell \rho _2^2-(1+2\rho _2)\omega _2\tanh ((L-\ell )/\omega _2)\} \\&\quad +\frac{|D|k}{2}(\mu _1(\ell )\mu _2(\ell ))^2 \left\{ \rho _2^2\left( \ell (1+\rho _1)^2-(\frac{3}{2}+2\rho _1)\omega _1\tanh (\ell /\omega _1)\right. \right. \\&\quad \left. +\frac{\ell }{2\cosh ^2(\ell /\omega _1)} \right) +\rho _1^2\left( (L-\ell )(1+\rho _2)^2-(\frac{3}{2}+2\rho _2)\omega _2\tanh ((L-\ell )/\omega _2)\right. \\&\quad \left. \left. +\frac{L-\ell }{2\cosh ^2((L-\ell )/\omega _2)} \right) \right\} \\&\quad +O(\beta _1^4)+O(\beta _2^4) \end{aligned} \end{aligned}$$

Note that by (C.2),

$$\begin{aligned} \rho _i=\beta _i/\gamma _i+O(\beta _i^2). \end{aligned}$$

Put $\widetilde{E}_s(\ell ):={{\mathcal {E}}}_s(\phi ,\psi )$. In view of (3.18) and (3.20), we clarify the terms with up to $O(|(\beta _1,\beta _2)|^2)$ in $\widetilde{E}_s(\ell )$ as

3.25 $$\begin{aligned} \begin{aligned} \widetilde{E}_s(\ell )&= \frac{|D|\beta _1}{2}\mu _1(\ell )^2 \{\ell (1+2\rho _1)-(1+2\rho _1)\omega _1\tanh (\ell /\omega _1)\} \\&+ \frac{|D|\beta _2}{2}\mu _2(\ell )^2 \{(L-\ell )(1+2\rho _2)-(1+2\rho _2)\omega _2\tanh ((L-\ell )/\omega _2)\} \\&+\frac{|D|k}{2}(\mu _1(\ell )\mu _2(\ell ))^2 \left\{ \rho _2^2 \left( \ell -\frac{3}{2}\omega _1\tanh (\ell /\omega _1) +\frac{\ell }{2\cosh ^2(\ell /\omega _1)} \right) \right. \\&\left. +\rho _1^2\left( (L-\ell )-\frac{3}{2}\omega _2\tanh ((L-\ell )/\omega _2) +\frac{L-\ell }{2\cosh ^2((L-\ell )/\omega _2)} \right) \right\} \\&+O(|(\beta _1,\beta _2)|^3)+O(\beta _1^4)+O(\beta _2^4). \end{aligned}\nonumber \\ \end{aligned}$$

With an appropriate choice of the parameters, the profile of $\widetilde{E}_s(\ell )$ is convex in an open interval of [0, *L*]. Thus, it is minimized by an $\ell$ in (0, *L*). Note that by (3.20), the approximation does not work near the boundaries $\ell =(0, L)$. It is clear that ${{\mathcal {E}}}_s(\phi ,\psi )=O(|(\beta _1,\beta _2)|)$ as $|(\beta _1,\beta _2)|\rightarrow 0$ under the restriction of (3.20). Since $\beta _i\rightarrow \alpha _i~(d_i\rightarrow 0)$, in view of (3.25), we have less energy nonconstant solution than any constant solution.

#### Proposition 3.7

Let $\Omega \subset \mathbb {R}^N~(1\le N\le 3)$ be a cylindrical domain as $\Omega =\{x=(x_1, x')\in (0,L)\times D\}$, where *D* is a bounded domain of $\mathbb {R}^{N-1}$ with smooth boundary. For the diffusion coefficients assume $d_i<D_i~(i=1,2)$. Then there are positive numbers $\overline{\alpha }$, $\overline{d}$ and $\overline{r}$ such that for

$$\begin{aligned} \alpha _i\le \overline{\alpha }, \quad d_i\le \overline{d}, \quad d_i/\alpha _i \le \overline{r} \quad (i=1,2), \end{aligned}$$

the system of (2.3)–(2.6) in $\Omega$ with (3.1) possesses a stable nonconstant equilibrium solution.

The energy (3.25) looks complicated. Here we consider the limiting behavior of (3.25) for the scaling as

3.26 $$\begin{aligned} \alpha _i=\varepsilon {\widetilde{\alpha }}_i, \quad d_i=\varepsilon ^{1+\delta }\widetilde{d_i}, \quad \delta >0\qquad (i=1,2), \end{aligned}$$

Then

3.27 $$\begin{aligned} \widetilde{e}(\ell ):=\lim _{\varepsilon \rightarrow 0}{{\mathcal {E}}}_s(\phi ,\psi )/\varepsilon =\frac{|D|}{2}\left\{ {\widetilde{\alpha }}_1\ell \left( \frac{Lm_1}{\ell }\right) ^2 +{\widetilde{\alpha }}_2(L-\ell )\left( \frac{Lm_2}{L-\ell }\right) ^2\right\} . \end{aligned}$$

A simple calculation shows that ${\widetilde{e}}(\ell )$ of (3.27) is minimized by

3.28 $$\begin{aligned} \ell ^*:=\frac{m_1\sqrt{{\widetilde{\alpha }}_1}}{m_1\sqrt{{\widetilde{\alpha }}_1}+m_2\sqrt{{\widetilde{\alpha }}_2}}L, \end{aligned}$$

at which

$$\begin{aligned} \widetilde{e}(\ell ^*)=\frac{|D|L}{2}(m_1\sqrt{{\widetilde{\alpha }}_1}+m_2\sqrt{{\widetilde{\alpha }}_2})^2. \end{aligned}$$

Consequently, we obtain

#### Corollary 3.8

Let $\varvec{u}_{\varepsilon }^*=({u_1^*}_\varepsilon , {u_2^*}_\varepsilon )$ be the minimizer of ${{\mathcal {E}}}_s$ with (3.26). Then

$$\begin{aligned} \limsup _{\varepsilon \downarrow 0}\frac{1}{\varepsilon }{{\mathcal {E}}}_\varepsilon (\varvec{u}_\varepsilon ^*)\le \frac{|\Omega |}{2} (m_1\sqrt{{\widetilde{\alpha }}_1}+m_2\sqrt{{\widetilde{\alpha }}_2})^2. \end{aligned}$$

This estimate might be useful for a mathematically rigorous study of the limiting behavior in the future work. Note that for the minimizer $\varvec{u}^*_\varepsilon$ of ${{\mathcal {E}}}_\varepsilon$

$$\begin{aligned} {{\mathcal {E}}}_\varepsilon (\varvec{u}^*_\varepsilon )\ge \frac{\varepsilon {\widetilde{\alpha }}_1}{2}\Vert u_{1\varepsilon }^*\Vert _{L^2}^2+\frac{\varepsilon {\widetilde{\alpha }}_2}{2}\Vert u_{2\varepsilon }^*\Vert _{L^2}^2 \ge \frac{\varepsilon |\Omega |}{2}({\widetilde{\alpha }}_1|\langle u_{1\varepsilon }^*\rangle ^2+{\widetilde{\alpha }}_2|\langle u_{2\varepsilon }^*\rangle |^2). \end{aligned}$$

We also expect that $(\phi , \psi )$ with $\ell =\ell _b$ gives a reasonable approximation of the minimizer of ${{\mathcal {E}}}_s$ in the parameter regime stated in Proposition 3.7, though $(\phi , \psi )$ is not smooth at $\ell =\ell _b$. As a matter of fact, the numerical test confirms that ${{\mathcal {E}}}_s$ has a minimal energy at $\ell _b=0.750762$ (Fig. 3b). Note that $\ell _b$ is corresponding to the location of the interface edge of pPAR domain boundary. When we define the edge of pPAR interface by the region where pPAR concentration is included in $(0.1\%, 0.5\%)$ of the minimal value of pPAR concentration in the full system (2.3)–(2.6) (Figs. 2c,  3a), the range of edge region of pPAR domain is approximated as a value in (0.745, 0.765), indicating that $\ell _b=0.750762$ gives a good approximation with respect to the location of polarity boundary. Fig. 3c also confirms that the approximate solution (3.21) for $\ell =\ell _b$ well captures the characteristic of the shape of polarity solution of the full system (2.3)–(2.6) shown in Fig. 3a. We will leave a mathematically rigorous proof of the minimizer of ${{\mathcal {E}}}_s$ in a future work because it is beyond the scope of this paper.

### Bulk-surface model and its shadow system

In this subsection, we show the existence of nonconstant equilibrium solutions for the shadow system (2.17)–(2.18).

As in §3.2, we define the following energy functional:

3.29 $$\begin{aligned} {{\mathcal {E}}}_1(u,v)&:=\int _{\Omega } \left\{ \frac{d_1}{2}|\nabla u|^2+\frac{d_2}{2}|\nabla v|^2+ \frac{\alpha _1}{2}u^2+\frac{\alpha _2}{2}v^2+\frac{k}{2} u^2v^2\right\} dx \nonumber \\&+\frac{\gamma _1(1-d_1/D_1)}{2}|\Omega |\left( M_1-\langle u\rangle \right) ^2+ \frac{\gamma _2(1-d_2/D_2)}{2}|\Omega |\left( M_2-\langle v\rangle \right) ^2. \end{aligned}$$

Then we can directly prove the existence of nonconstant equilibrium solution for (2.17)–(2.18) with (2.19), provided that the system has the scaled parameters (2.10) with the condition in Proposition 3.7. Numerical simulations also confirm that nonconstant equilibrium solutions exist for both Neumann and periodic boundary conditions (Fig. 2d).

As seen in the derivation of the shadow system from the bulk-surface model, it is reasonable to consider the shadow system in a 2-dimensional closed surface of the cell membrane. It is also interesting to study how the geometry of the surface affects the spatial pattern. Those issues are beyond the present scope and will be good as a future work. However, we expect that the above observation in a general domain will be useful for a further study in various domains.

#### Remark 3.2

In Goehring et al. (2011b), the following model equations for plolarization of PAR proteins are proposed:

3.30 $$\begin{aligned}&\partial _tA+\partial _x(\nu A)=D_{\mathrm {A}}\partial _x^2A-k_{\mathrm {off, A}}A-k_{\mathrm {AP}}P^\alpha A+k_{\mathrm {on, A}}A_{\mathrm {cyto}}, \end{aligned}$$

3.31 $$\begin{aligned}&\partial _tP+\partial _x(\nu P)=D_{\mathrm {P}}\partial _x^2P-k_{\mathrm {off, P}}P-k_{\mathrm {AP}}A^\beta P+ k_{\mathrm {on, P}}P_{\mathrm {cyto}}, \end{aligned}$$

where *A* and *P* stand for the local membrane concentrations of aPARs and pPARs, respectively. On the other hand, $A_{\mathrm {cyto}}$ and $P_{\mathrm {cyto}}$ stand for the uniform cytoplasmic concentrations of aPARs and pPARs, respectively, that is, those are assumed to be constants. In this model, they set a bistable character of the system, that is, the system allows two stable spatially uniform steady states by tuning parameters suitably. Goehring et al. (2011b) shows that the cortical flow velocity, $\nu =\nu (x,t)$, plays a role in serving as a mechanical trigger for the pattern formation in the initial stage and the bistable character plays to create a segregation pattern of *A* and *P* after the flow effect ceases. However, when $\nu =0$, there is no stable equilibrium state because the system with $\nu =0$ belongs to a class of competition-diffusion systems. In fact, Kishimoto (1981) and Kishimoto and Weinberger (1985) show the instability of nonconstant equilibrium solutions of two component reaction–diffusion equations of competition type in an interval or a convex domain with the Neumann boundary condition or periodic boundary condition. On the other hand, by virtue of the nonlocal constraint of (2.16), the system (2.17)–(2.18) allows a stable nonconstant equilibrium in a parameter regime as seen in §3.3. Although the mathematical theorem ensures non-existence of stable segregation patterns in the system (3.30)–(3.31) with $\nu =0$, a very slow motion of the transition layer might happen in Goehring et al. (2011b), as seen in the scalar reaction–diffusion equation by Car and Pego (1989) and Fusco and Hale (1989).

**Fig. 4:**
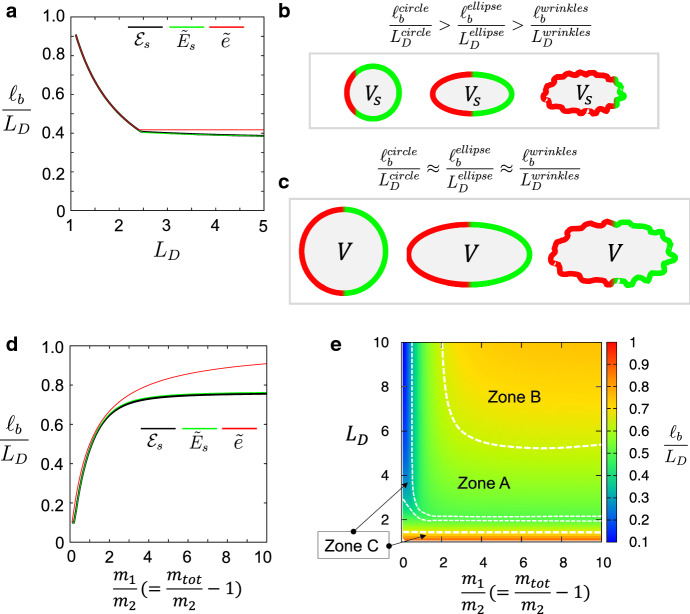
The size of polarity domain and the effect of cell shape. **a** The effect of the cell membrane length on the size of the pPAR domain is plotted by the energy functionals ${{\mathcal {E}}}_s$, ${\widetilde{E}}_{s}$, and ${\widetilde{e}}$ ((3.24), (3.25), and (3.27)). Here, $L_D=L/|D|$ where |*D*| is fixed. In addition, $\ell _b$ is the length of the pPAR domain corresponding to the value of $\ell$ where the energy functionals (3.24), (3.25), and (3.27) have minimal energy, respectively. **b**–**c** Schematic figures for the effect of the cell shape to the length scale (boundary position) of the polarity domain. From **a**, the ratio of the pPAR (aPAR) domain to the cell membrane length becomes constant. $V_S$ and *V* are the cell volumes and $Vs\ll V$. $L_D^{name}$ is the length of cell circumference. **d** The effect of the total mass on the size of the pPAR domain is plotted by the energy functionals. **E** The effects of both the cell membrane length and the total mass. The same parameter values as in Fig. 2 are chosen for **a**, **d**, and **e**

### Energy functional and biological implication

The length of the PAR polarity domain in asymmetric cell division is considered an important factor in regulating the location of the cell cleavage plane during asymmetric cell division (Coffmana et al. 2016; Morton et al. 2002; Rose and Kemphues 1998). However, the mechanism by which the polarity length is determined remains elusive, although this issue has been partially noted in both experimental and theoretical studies (Dawes and Iron 2013; Goehring et al. 2011b; Seirin-Lee and Shibata 2015; Seirin-Lee 2020). Herein, we explore how the size of polarity domains (namely, the position of the polarity boundary) is affected by the size of the cell membrane (*L*) and the total mass of the polarity proteins by using the energy functionals obtained in Sect. 3.3.

We first explore how the boundary location of the pPAR polarity (namely, $\ell _b$) is determined, which also defines the relative size of a polarity domain with respect to the size of the cell membrane. The boundary location of the aPAR polarity is be given to $L-\ell _{b}$. In the model systems (2.3)–(2.6) for $\Omega =(0, L)\times D$, *L* can be regarded as the cell circumference of the cell membrane and |*D*| is the membrane thickness. We thus fix the size of |*D*| and vary *L*. We calculated $\ell _b$ with respect to $L_D=L/|D|$ by using the energy functionals, (3.24), (3.25), and (3.27), the results of which are shown in Fig. 4a. We first found that when the size of the cell membrane is relatively small (i.e. in a small cell), the size of the pPAR domain decreases as the length of the cell membrane increases. This suggests that the length of the polarity can be critically affected by the shape of the cell (Fig. 4a, b). Because the circumference of the cell membrane in an elliptic cell is larger than that in a circular cell under the same volume, a circular cell has a longer domain of pPAR than the elliptic cell. That is, we have $\frac{\ell _b^{circle}}{L_D^{circle}}>\frac{\ell _b^{ellipse}}{L_D^{ellipse}}$ because $L_D^{circle}<L_D^{ellipse}$ and $\ell _b^{circle}>\ell _b^{ellipse}$ (Fig. 4b). By contrast, when the volume of the cell is large, namely the length of the cell membrane is large, the relative domain size of pPAR becomes constant without a dependence on $L_D$ (Fig. 4a, c). This suggests that the boundary position of the polarity is determined very robustly without the sensitivity of the cell shape. This fact is likely to be held for any cell shape, such as in the case of wrinkled cells (Fig. 4b, c).

Next, we investigate the effect of the total mass. We fix the total mass of aPAR ($m_2$) and vary the total mass of pPAR ($m_1$), which consequently leads to a variation of the total masses of pPAR and aPAR ($m_{tot} = m_1 + m_2$) because $m_1/m_2 = (m_{tot}/m_2-1)$. As $m_1$ (or $m_{tot}$) increases, the length of the polarity domain is increased (Fig. 4d). Unexpectedly, however, we found that once the total mass becomes larger than a certain amount, the length of the polarity domain is constant despite the increase in the total mass. This implies that if the total mass is sufficiently large, it may not be a dominant factor in determining the polarity length, while the other kinetic parameters or elements are likely to play an important role.

Finally, we explored both the effects of the length of the cell membrane ($L_D$) and the total mass of the polarity proteins on the length of the polarity domain (Fig. 4e). We found that a proper relation between the total mass and the cell membrane length is required to obtain a proper size of the polarity domain (a proper position of the polarity boundary) (Zone A). Specifically, if the length of the cell membrane is too small or the total mass is insufficient, the polarity domain either spreads out to a wide region of the cell membrane or remains in an area with a small polarity (Zone C). We also found that a wide parameter region exists where the sensitivity of $L_D$ and the ratio of the total mass to the size of the polarity domain becomes negligible (Zone B). Note that, although the reduced energy functional ${\widetilde{e}}$ in (3.27) does not show a good approximation quantitatively compared to the other two energy functionals ${{\mathcal {E}}}_s$ in (3.24) and ${\widetilde{E}}_{s}$ in (3.25) (Fig. 4a, d), it still captures well a qualitative characteristic of the nonconstant stationary solution. Thus, we can directly obtain similar conclusions with the Eq. (3.28).

## Discussion

The pattern formation system characterised by bi-stability and mass conservation has been highlighted using a cell polarity model and explored based on several kinetic types of models (Goehring et al. 2011b; Mori et al. 2008; Otsuji et al. 2007; Seirin-Lee and Shibata 2015; Seirin-Lee 2021; Trong et al. 2014). In this study, we have explored the long time behavior of stable patterns for high-dimensional polarity models describing the PAR polarity occurring in asymmetric cell division, which are based on the models suggested by Goehring et al. (2011b) and Seirin-Lee and Shibata (2015). We showed that the long time behavior around the stationary solution in the bulk-surface model can be understood by reduced models; a cell membrane periphery model and shadow system have a mathematically simpler form but are able to capture the dynamics of the stationary polarity solutions in a high-dimensional space, and we rigorously established the existence and stability of the polarity solutions.

In this study, we also succeeded in constructing a reasonable approximate solution that provides a direct calculation of the energy functionals. Using these energy functionals, we have found the detailed effect of the cell membrane length and total mass on determining the size of polarity domain (the position of the polarity boundary). From this analysis, we also found that the size of the polarity domain can be sensitively altered depending on the cell shape when the cell size is small. By contrast, the relative location of the boundary is not changed by the cell shape when the cell size is large, which implies that a bigger cell may need to have fewer factors to maintain a robust length of the polarity domain than a smaller cell.

A previous study by Seirin-Lee and Shibata (2015) has found that the pPAR polarity length is linearly dependent on the total mass. However, their observations were restricted to a specific parameter range because the total mass was not an independent parameter but was determined based on the choice of kinetic parameters. A numerical analysis carried out on full partial-differential equations with respect to multiple parameter values often limits the computations in terms of the cost of the numerical calculation, though the recent study on the numerical method and scheme shows the development (Hao and Xue 2020; Uecker et al. 2014). By contrast, we can deal with the total mass as an independent parameter in our analysis using the energy functionals. This provides a highly effective numerical cost when we investigate the effects of the parameters on the dynamics of the solutions with a polarity profile.

In this study, our main approach to the model reductions has been based on the assumption of homogeneous state of cytosol protein which is basically resulted from a fast diffusion in the cytosol space. The approximation of shadow system requires a mathematical condition of diffusion infinity in the cytosol ($D_c\rightarrow \infty$), but it is not a biologically feasible condition. On the other hand, the cell membrane periphery model does not require such a limit condition and we can obtain mathematically rigorous results under the biologically reasonable condition that the diffusion in the cytosol is faster than that on the membrane $(D_c>D_m)$. This is generally observed in a cell (Kuhn et al. 2011). Thus, our analytical approach to the shadow system via a cell membrane periphery model supports that the mathematical assumption of fast diffusion in the cytosol is reasonable to understand the essential structure of polarity formation.

Although our mathematical analysis captures well the dynamics of the polarity in qualitative terms, it would be interesting to confirm the effect of the cell geometry on the length of the polarity domain in real biological systems. We are currently considering this, and leave it as a subject of future study.

## Figures and Tables

**Fig. 5:**
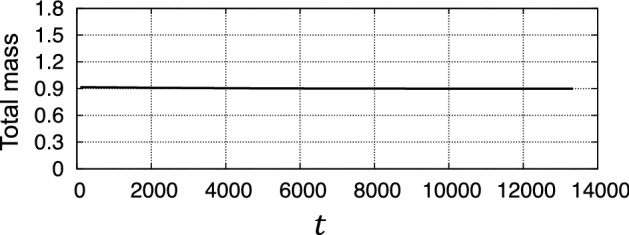
Conservation of total mass in phase-field-combinded model. The concentration of total mass of aPAR and pPAR is plotted for the model (1.2) with (1.4) calculated in Fig. 2a. The average of numerical data of the total mass is 0.90319596, and the standard derivation is 0.00474245

## Modeling method and numerical information

### Bulk-surface modeling using phase-field method

To describe the bulk-cytosol space and the membrane surface, we used a method of combining a phase-field function with the reaction–diffusion system. We apply the method proposed by Seirin-Lee (2016a, 2017) with respect to the case of a single cell. We solved the following equation in order to generate a cell shape:

A.1 $$\begin{aligned} \mu ^{-1}\frac{\partial \phi }{\partial t}=\varepsilon ^2\nabla ^2\phi +\phi (1-\phi )\left\{ \phi -\frac{1}{2}-60\alpha \phi (1-\phi )\left( V(t)-\overline{V}\right) \right\} , \end{aligned}$$

where $\alpha (>0)$ is the intensity constant of the energy for the volume *V*(*t*), and $\overline{V}$ is the target volume of the cell. With a phase-field function satisfying (A.1) for some time $t^*$, $\phi (\mathbf {x},t^*)$, we define a fixed cell domain as follows :

$$\begin{aligned}&\text {Cytosol}\equiv \{\mathbf {x}|\phi (\mathbf {x},t^*)=1 \}, \quad \text {Membrane}\equiv \{\mathbf {x}|0<\phi (\mathbf {x},t^*)<1\},\\&\text {Extracellular region}\equiv \{\mathbf {x}|\phi (\mathbf {x},t^*)=0\}, \end{aligned}$$

where $$\mathbf {x}\in \mathbb {R}^N$$.

In next, we combine the bulk-surface model (1.2) with the cell phase-field function $\phi (\mathbf {x},t^*)$ (Kockelkoren et al. 2003; Levine and Rappel 2005; Wang et al. 2017). The model system combined with the phase-field function which we used for the numerical simulations of bulk-surface model (1.2) is given to

A.2 $$\begin{aligned} \begin{aligned}&\frac{\partial B(\phi )P_{m}}{\partial t}= D^{P}_{m}\nabla \cdot (B(\phi )\nabla P_{m})+B(\phi )\{ -F_{\text {off}}(A_m){P_m}+\gamma {P_c} \}, \\&\frac{\partial \phi P_{c}}{\partial t} = D^{P}_{c}\nabla \cdot (\phi \nabla P_{c})+|\nabla \phi |\{ F_{\text {off}}(A_m){P_m}-\gamma {P_c} \}, \\&\frac{\partial B(\phi ) A_{m}}{\partial t} = D^{A}_{m}\nabla \cdot (B(\phi )\nabla A_{m})+B(\phi )\{ -\overline{F}_{\text {off}}(P_m){A_m}+{\overline{\gamma }}{A_c} \},\\&\frac{\partial \phi A_{c}}{\partial t} = D^{A}_{c}\nabla \cdot (\phi \nabla A_{c} )+|\nabla \phi |\{ \overline{F}_{\text {off}}(P_m){A_m}-{\overline{\gamma }}{A_c} \}, \end{aligned} \end{aligned}$$

where $B(\phi )=\nu \phi ^{2}(1-\phi )^2~(\nu >0)$, a function defining the membrane region.

Because the cell shape can be easily generated by the Eq.  (A.1) by adjusting the target volume $\overline{V}$ and initial condition, the phase-field-combinded modeling is very convenient to simulate various cell shapes without lengthy numerical implementation. To make a cell, we used the parameter values: $\alpha =120,~\overline{V}=0.2828, ~\mu =1.0,~\varepsilon =2.0\times 10^{-3}, ~\nu =16.0$, and

$$\begin{aligned} \phi (\mathbf {x}, 0)=\left\{ \begin{array}{cc}1 &{} \text {in} ~~\{(x,y)| \frac{(x-0.5)^2}{0.225^2}+\frac{(y-0.5)^2}{0.400^2}\le 1 \}, \\ 0 &{} \text {otherwise,}\end{array}\right. \end{aligned}$$

on $$\mathbf {x}\in [0, 1]\times [0, 1]$$.

### Numerical scheme and stability

We solved the form of the bulk-surface system (A.2) using C language by a standard explicit numerical scheme on square grids. The system domain is given to $[0, 1]\times [0, 1]$ and has been spaced by $400\times 400$ grids which defines $\triangle x=0.0025$. $\triangle t=1/300$ is chosen so that the stability condition of explicit scheme

$$\begin{aligned} \text {(Maximal diffusion coefficient)}\times \frac{\triangle t }{(\triangle x)^2}=0.00036\times \frac{1/300}{0.0025}=0.00048<\frac{1}{2} \end{aligned}$$

is sufficiently satisfied (Morton and Mayers 1994). We also have confirmed that the simulation results are not changed with smaller spatial and temporal grid size. Figure S5 confirms that the system (A.2) combined with phase-field functions is well-conserved numerically with respect to the total mass.

On the other hand, we solved one and two dimensional reaction–diffusion systems with Neumann and periodic boundary conditions by implicit method using LU decomposition and ADI method (Morton and Mayers 1994).

### Initial conditions

The initial condition for simulating the bulk-surface model (1.2) was given to locally concentrated initial conditions (Seirin-Lee and Shibata 2015) by

$$\begin{aligned}&A_{m}(\mathbf {X},0)=A_{m}^0(1+\delta \psi (\mathbf {X}))&\text {on}&\ \mathbf {X}\in \partial \Omega , \\&A_{c}(\mathbf {X},0)=A_{c}^0(1+\delta \psi (\mathbf {X}))&\text {on}&\ \mathbf {X}\in \Omega , \\&P_{m}(\mathbf {X},0)=\sigma&\text {on}&\ \mathbf {X}\in \omega \subset \partial \Omega , \\&P_{m}(\mathbf {X},0)=0&\text {on}&\ \mathbf {X}\in \partial \Omega \setminus \omega , \\&P_{c}(\mathbf {X},0)=P_{c}^{0}(1+\delta \psi (\mathbf {X}))&\text {on}&\ \mathbf {X}\in \Omega , \end{aligned}$$

where $\sigma$ is the strength of the signal, and $\omega$ is the sufficiently small region in which the signal is imposed. $\delta$ is a positive constant and has a very small value. Typically, we set $\sigma =40$ with $|\omega |=B(\phi )(0.05)^2$ and $\delta =0.001$. $\psi (\mathbf {X})$ is a random function of uniform distribution, which takes values in the range of $[-0.5, 0.5]$. The detailed values used in the representative simulations are $A_{m}^0=P_{m}^0=1.02,$

$$\begin{aligned} A_{c}^0=\frac{1}{{\bar{\gamma }}}\left( {\bar{\alpha }}+ \frac{\overline{K}_1(P_m^0)^2}{1+\overline{K}(P_m^0)^2} \right) A_m^0, \quad \text {and}\quad P_{c}^0=\frac{1}{\gamma }\left( \alpha + \frac{{K}_1(A_m^0)^2}{1+{K}(A_m^0)^2} \right) P_m^0. \end{aligned}$$

## Proof of Lemmas in Section 3

### Proof of Lemmas 3.1, 3.2 and 3.3

#### Proof of Lemma 3.1

We first consider

$$\begin{aligned}&\partial _t{\widetilde{u}_1}=d_1\Delta {\widetilde{u}_1}-(\alpha _1+k \widetilde{u}_2^2){\widetilde{u}_1}+\gamma _1\widetilde{v}_1, \\&\partial _t{\widetilde{v}_1}=D_1\Delta {\widetilde{v}_1}+(\alpha _1+k \widetilde{u}_2^2)(\widetilde{u}_1)_{+}-\gamma _1\widetilde{v}_1, \\&\tau \partial _t{\widetilde{u}_2}={d}_2\Delta {\widetilde{u}_2}-(\alpha _2+k \widetilde{u}_1^2){\widetilde{u}_2}+\gamma _2 {\widetilde{v}_2}, \\&\tau \partial _t{\widetilde{v}_2}={D}_2\Delta {\widetilde{v}_2}+(\alpha _2+k \widetilde{u}_1^2)(\widetilde{u}_2)_{+}-\gamma _2{\widetilde{v}_2}, \end{aligned}$$

in $\Omega$ with the same initial condition, where $(\widetilde{u}(x,\cdot ))_{+}:=\max \{\widetilde{u}(x,\cdot ), 0\}$. This system has a unique classical solution in $\overline{\Omega }\times [0,T )$ for some $T>0$ [see Rothe (1984)]. Since

$$\begin{aligned} \partial _t{\widetilde{v}_1}\ge D_1\Delta {\widetilde{v}_1}-\gamma _1\widetilde{v}_1, \qquad \tau \partial _t{\widetilde{v}_2}\ge {D}_2\Delta {\widetilde{v}_2}-\gamma _2{\widetilde{v}_2}, \end{aligned}$$

we use the maximum principle to assert $\widetilde{v}_1(x,t), \widetilde{v}_2(x,t)\ge 0 ~(0\le t<T)$. This leads us $\widetilde{u}_1(x,t), \widetilde{u}_2(x,t)\ge 0~(0\le t <T)$ by applying the maximum principle to the $\widetilde{u}_1$ and $\widetilde{u}_2$ equations. The strong maximum principle shows the strict positivity of the solutions for $t>0$. In the sequence the solutions, $({u}_1, {u}_2, {v}_1, {v}_2)$ and $(\widetilde{u}_1, \widetilde{u}_2, \widetilde{v}_1, \widetilde{v}_2)$ coinside in $\overline{\Omega }\times [0,T )$ because of the uniqueness of the solution. $\square$

#### Proof of Lemma 3.2

We let ${{\mathcal {E}}}(\varvec{u}, \varvec{z})$ be the function defined in (3.7). For a solution $(u_1(\cdot , t), v_1(\cdot , t), u_2(\cdot , t), v_2(\cdot , t))$ to the system (2.3)–(2.6) and

$$\begin{aligned} z_1(\cdot , t)=(d_1/D_1)u_1(\cdot , t)+v_1(\cdot , t),\qquad z_2(\cdot , t)=(d_2/D_2)u_2(\cdot , t)+v_2(\cdot t), \end{aligned}$$

we compute

$$\begin{aligned}&\frac{d}{dt} {{\mathcal {E}}}(\varvec{u}(\cdot , t), \varvec{z}(\cdot , t)) \\&\quad =\int _{\Omega } [d_1\nabla u_1\cdot \nabla (\partial _t u_1) +d_2\nabla u_2\cdot \nabla (\partial _t u_2) +(\alpha _1+\gamma _1d_1/D_1)u_1\partial _t u_1\\&\qquad +(\alpha _2+\gamma _2d_2/D_2)u_2\partial _t u_2 +k u_2^2 u_1\partial _t u_1 +ku_1^2u_2{\partial _t} u_2\\&\qquad +\theta _1z_1\partial _t z_1+\theta _2z_2\partial _t z_2 ]dx\\&\quad =\int _{\Omega } [\{-d_1\Delta u_1+\alpha _1 u_1+(\gamma _1d_1/D_1)u_1+ku_2^2u_1\}\partial _t u_1\\&\qquad +\{-d_2\Delta u_2+\alpha _2 u_2+(\gamma _2d_2/D_2)u_2+ku_1^2u_2\}\partial _t u_2 \\&\qquad +\theta _1z_1(-(1-d_1/D_1)\partial _t{u_1}+D_1\Delta z_1)\\&\qquad +\theta _2(-(1-d_2/D_2)\partial _t{u_2}+(1/\tau )D_2\Delta z_2)]dx\\&\quad =\int _{\Omega } [(-\partial _t u_1+\gamma _1z_1)\partial _t u_1+(-\tau \partial _t u_2+\gamma _2z_2)\partial _t u_2 \\&\qquad -\gamma _1z_1\partial _t{u_1}-\theta _1D_1|\nabla z_1|^2 -\gamma _2z_2\partial _t{u_2}-(\theta _2/\tau )D_2|\nabla z_2|^2]dx \\&\quad =-\int _{\Omega }[(\partial _t u_1)^2+\tau (\partial _t u_2)^2+\theta _1D_1|\nabla z_1|^2+(\theta _2/\tau )D_2|\nabla z_2|^2]dx. \end{aligned}$$

This implies

$$\begin{aligned}&{{\mathcal {E}}}(\varvec{u}(\cdot , t), \varvec{z}(\cdot , t)) +\int _0^t\left\{ \int _{\Omega }[(\partial _t u_1)^2+\tau (\partial _t u_2)^2+\theta _1D_1|\nabla z_1|^2 \right. \\&\qquad \left. +(\theta _2/\tau )D_2|\nabla z_2|^2]dx\right\} dt' \\&\quad \le {{\mathcal {E}}}(\varvec{u}(\cdot , 0), \varvec{z}(\cdot , 0)). \end{aligned}$$

Put

$$\begin{aligned} \beta _i:=\alpha _i+\gamma _id_i/D_i \quad (i=1,2). \end{aligned}$$

Since

$$\begin{aligned} \frac{1}{2}\min \{ d_1, d_2, \beta _1, \beta _2, \theta _1, \theta _2\}(\Vert u_1\Vert _{H^1}^2+\Vert u_2\Vert _{H^1}^2+\Vert z_1\Vert _{L^2}^2+\Vert z_2\Vert _{L^2}^2) \le {{\mathcal {E}}}(\varvec{u}, \varvec{z}), \end{aligned}$$

the assertion of the lemma immediately follows. $\square$

Before going to the proof of Lemma 3.3, we prepare some estimate for the heat equation. Let $u(\cdot , t; u_0)$ be a unique solution of

$$\begin{aligned} \partial _t u=d\Delta u~~\mathrm {in}~~\Omega , \quad \partial u/\partial \varvec{n}=0~\mathrm {on}~\partial \Omega , \quad u(\cdot ,0)=u_0~(\in C(\overline{\Omega }; \mathbb {R})), \end{aligned}$$

and define the the semigroup $\{e^{td\Delta }\}_{t\ge 0}$ by $e^{td\Delta }u_0:=u(\cdot , t; u_0)$. We will use the following inequality ( Rothe (1984)):

B.1 $$\begin{aligned} \Vert e^{td\Delta }\phi \Vert _{L^r}\le C_0(d, q,r)\max \{ 1, t^{-\frac{N}{2}(\frac{1}{q}-\frac{1}{r})}\}\Vert \phi \Vert _{L^q}, \quad 1\le q\le r\le \infty . \end{aligned}$$

#### Proof of Lemma 3.3

In view of $\langle u_i(\cdot ,t)\rangle +\langle v_i(\cdot ,t)\rangle =m_i$ and the positivity of the solution, we see $\langle u_i(\cdot ,t)\rangle , \langle v_i(\cdot ,t)\rangle \le m_i~(t\ge 0)$ for $i=1,2$. Thus, $\langle z_i(\cdot , t)\rangle \le (1+d_i/D_i)m_i~(t\ge 0)$ for $i=1,2$. Then, there is a positive $C_2$ such that for $i=1,2$

B.2 $$\begin{aligned} \Vert z_i(\cdot , t)\Vert _{L^2}^2=\Vert \langle z_i(\cdot ,t) \rangle \Vert _{L^2}^2+\Vert z_i(\cdot ,t)-\langle z_i(\cdot ,t)\rangle \Vert _{L^2}^2 \le C_2(1+\Vert \nabla z_i(\cdot ,t)\Vert ^2_{L^2}) \end{aligned}$$

holds.

In the rest of the proof, we omit to state the boundary condition of the equations since it is fixed. We prove the assertion of the lemma for only $\tau =1$ below since the same argument can be easily applied to the case $\tau \ne 1$ with a slight modification.

When $N=1$, since $H^1(\Omega )\subset C^{1/2}(\overline{\Omega })$, we obtain the uniform boundedness for $u_i(x,t)$. The solution $\overline{v}_i(\cdot ,t)$ of

B.3 $$\begin{aligned} \partial _t\overline{v}_i=D_i\Delta \overline{v}_i-\gamma _i \overline{v}_i +(\alpha _i+kC^2)C, \end{aligned}$$

with $\overline{v}_i(\cdot ,0)=v_{i,0}(\cdot )$, bounds $v_i(\cdot , t)$ by the maximum principle. Hence, we easily see the uniform boundedness for $v_i(\cdot ,t)$ for $i=1,2$.

For $N\ge 2$, taking $q=2$ and *r* with $1\le r<2N/(N-2)_{+}$, we have

$$\begin{aligned} \frac{N}{2}(\frac{1}{2}-\frac{1}{r})<\frac{1}{2}, \end{aligned}$$

and by (B.1)

$$\begin{aligned} \Vert e^{td\Delta }\phi \Vert _{L^r}\le C_0\max \{ 1, t^{-\frac{N}{2}\left( \frac{1}{2}-\frac{1}{r}\right) }\}\Vert \phi \Vert _{L^2} \end{aligned}$$

(precisely, $C_0=C_0(d,2,r)$, but we omit the dependence on *d* and *r*). The equations of (3.4) and (3.6) allow the integral form as

$$\begin{aligned} z_i(\cdot ,t)=e^{t(D_i\Delta -1)}z_i(\cdot ,0)+\int _0^t e^{(t-t')(D_i\Delta -1)}\{z_i(\cdot , t')-(1-d_i/D_i)\partial u_i(\cdot , t')\}dt', \end{aligned}$$

for $i=1,2$. In view of (B.2), (3.8) and the uniform boundedness of the initial data, there is a positive constant $C_3$ such that

$$\begin{aligned}&\Vert z_i(\cdot ,t)\Vert _{L^r} \le C_3\Vert z_i(\cdot ,0)\Vert _{L^2} \\&\quad + C_3 \int _0^t \{ (t-t')^{-\frac{N}{2}(\frac{1}{2}-\frac{1}{r})}e^{-(t-t')}(\Vert z_i(\cdot ,t')\Vert _{L^2}+ \Vert \partial _t u_i(\cdot , t')\Vert _{L^2}\}dt' \\&\quad \le C_3\Vert z_i(\cdot ,0)\Vert _{L^2} + C_3\sqrt{{C_2}}\int _0^t (t-t')^{-\frac{N}{2}(\frac{1}{2}-\frac{1}{r})}e^{-(t-t')}dt' \\&\quad +C_3\left( \int _0^t (t-t')^{-N(\frac{1}{2}-\frac{1}{r})} e^{-2(t-t')}dt'\right) ^{1/2} \\&\qquad \times \left( 2\int _0^t ({C_2}\Vert \nabla z_i(\cdot ,t')\Vert _{L^2}^2+ \Vert \partial _t u_i(\cdot , t')\Vert _{L^2}^2) dt'\right) ^{1/2}. \end{aligned}$$

Consequently, for $$1\le r<2N/(N-2)_{+}$$, there is $$ C_4>0$$ such that

B.4 $$\begin{aligned} \Vert z_i(\cdot ,t)\Vert _{L^r} \le C_4. \end{aligned}$$

Since the solution $u_i$ is bounded by a solution $\overline{u}_i$ to

B.5 $$\begin{aligned} \partial _t\overline{u}_i=d_i\Delta \overline{u}_i-\alpha _i \overline{u}_i+\gamma _i z_i(x,t), \end{aligned}$$

with the same initial condition, we estimate the solution to (B.5). We write (B.5) in the integral form as

B.6 $$\begin{aligned} \overline{u}_i(\cdot ,t)=e^{t(d_i\Delta -\alpha _i)}\overline{u}_i(\cdot , 0) +\gamma _i\int _0^t e^{(t-t')(d_i\Delta -\alpha _i)} z_i(\cdot ,t')\ dt'. \end{aligned}$$

We notice that the inequality

$$\begin{aligned} \frac{N}{2}\frac{1}{r}<\frac{1}{2} \end{aligned}$$

leads to $N<r<2N/(N-2)_{+}$, so $(N-2)_{+}<2$. Thus, for $N=3$, by taking $q=5$ and $r=\infty$ in (B.1), we have

$$\begin{aligned} \Vert e^{td\Delta }\phi \Vert _{L^{\infty }}\le C_0\max \{ 1, t^{-\frac{3}{10}}\}\Vert \phi \Vert _{L^5}. \end{aligned}$$

Applying this to (B.6) yields that there is $$ C_5>0$$ such that

$$\begin{aligned} \Vert \overline{u}_i(\cdot ,t)\Vert _{L^{\infty }}\le C_5\Vert u_{i,0}\Vert _{L^{\infty }}+C_5\int _0^t (t-t')^{-\frac{3}{10}} e^{-\alpha _i(t-t')}\Vert z_i(\cdot ,t')\Vert _{L^5}dt' \qquad (\forall t>0). \end{aligned}$$

In terms of (B.4), the uniform boundedness of $\Vert u_i(\cdot ,t)\Vert _{L^\infty }$ follows.

In order to obtain the uniform boundedness of $v_i$, we consider (B.3) and make use of the maximum principle again.

As for $N=2$, we can take any *q* greater than 2 and $r=\infty$ in (B.1). A similar argument leads to the desired assertion. $\square$

### Proof of Lemma 3.5

#### Proof of Lemma 3.5

In view of ${{\mathcal {E}}}(\varvec{u}^*, \varvec{z}^*)={{\mathcal {E}}}_s(\varvec{u}^*)$ and (3.14), we have

B.7 $$\begin{aligned} {{\mathcal {E}}}_s(\varvec{u}(\cdot ,t))-{{\mathcal {E}}}_s(\varvec{u}^*)&\le {{\mathcal {E}}}(\varvec{u}(\cdot ,t), \varvec{z}(\cdot ,t))-{{\mathcal {E}}}(\varvec{u}^*, \varvec{z}^*)\nonumber \\&\le {{\mathcal {E}}}(\varvec{u}(\cdot ,0), \varvec{z}(\cdot ,0))-{{\mathcal {E}}}(\varvec{u}^*, \varvec{z}^*)\qquad (t\ge 0). \end{aligned}$$

Let $\varepsilon _1$ be as in Lemma 3.4. Given $\varepsilon \in (0,\varepsilon _1/4]$, we let $\delta _1=\delta _1(\varepsilon )$ be same as in Lemma 3.4. Take $\delta >0$ so that

B.8 $$\begin{aligned}&\Vert \varvec{u}(\cdot ,0)-\varvec{u}^*\Vert _{H^1}<\varepsilon _1/2\quad \text{ and }\quad {{\mathcal {E}}}(\varvec{u}(\cdot ,0), \varvec{z}(\cdot ,0))-{{\mathcal {E}}}(\varvec{u}^*, \varvec{z}^*)<\min \{\delta _1,\varepsilon ^2 \},\nonumber \\&\mathrm {if}\quad \Vert (\varvec{u}(\cdot ,0), \varvec{z}(\cdot ,0))-(\varvec{u}^*, \varvec{z}^*)\Vert _{H^1}<\delta . \end{aligned}$$

In view of (B.7), we have

B.9 $$\begin{aligned} {{\mathcal {E}}}_s(\varvec{u}(\cdot ,t))-{{\mathcal {E}}}_s(\varvec{u}^*)<\delta _1\quad (t\ge 0). \end{aligned}$$

In addition we have

B.10 $$\begin{aligned} \Vert \varvec{u}(\cdot ,t)-\varvec{u}^*\Vert _{H^1}<\varepsilon _1/2\qquad (t\ge 0). \end{aligned}$$

Indeed, if there is $t_1>0$ such that

$$\begin{aligned} \Vert \varvec{u}(\cdot ,t_1)-\varvec{u}^*\Vert _{H^1}=\varepsilon _1/2, \end{aligned}$$

then a contradiction follows from

$$\begin{aligned} \Vert \varvec{u}(\cdot ,t_1)-\varvec{u}^*\Vert _{H^1}<\varepsilon \le \varepsilon _1/4, \end{aligned}$$

by the assertion of Lemma 3.4.

Consequently, by (B.9) and (B.10), Lemma 3.4 gives

B.11 $$\begin{aligned} \Vert \varvec{u}(\cdot ,t)-\varvec{u}^*\Vert _{H^1}<\varepsilon \qquad (t\ge 0). \end{aligned}$$

On the other hand, by (B.7) and (B.8)

$$\begin{aligned}&\varepsilon ^2\ge {{\mathcal {E}}}(\varvec{u}(\cdot ,0), \varvec{z}(\cdot ,0))-{{\mathcal {E}}}(\varvec{u}^*, \varvec{z}^*)\ge {{\mathcal {E}}}(\varvec{u}(\cdot ,t), \varvec{z}(\cdot ,t))-{{\mathcal {E}}}(\varvec{u}^*, \varvec{z}^*)\\&\quad ={{\mathcal {E}}}_s(\varvec{u}(\cdot ,t))-{{\mathcal {E}}}_s(\varvec{u}^*)+\frac{\theta _1}{2}\Vert \nabla z_1(t)\Vert ^2_{L^2}+\frac{\theta _2}{2}\Vert \nabla z_2(t)\Vert ^2_{L^2}, \end{aligned}$$

which yields

$$\begin{aligned} \Vert \nabla \varvec{z}(\cdot ,t)\Vert _{L^2}^2\le \frac{2\varepsilon ^2}{\min \{\theta _1,\theta _2\}}. \end{aligned}$$

We estimate

$$\begin{aligned} |\langle z_i(\cdot ,t)\rangle - \langle z_i^*\rangle |^2\le |\langle u_i(\cdot ,t)\rangle - \langle u_i^*\rangle |^2 \le \frac{1}{|\Omega |}\Vert u_i(\cdot ,t) - u_i^*\Vert _{L^2}^2, \end{aligned}$$

and

$$\begin{aligned} \Vert \varvec{z}(\cdot ,t)-\langle \varvec{z}(\cdot , t)\rangle \Vert _{L^2}^2\le C_1\Vert \nabla \varvec{z}(\cdot ,t)\Vert _{L^2}^2. \end{aligned}$$

In the sequence, we obtain

$$\begin{aligned} \Vert \varvec{z}(\cdot ,t)-\varvec{z}^* \Vert _{H^1}^2&\le \Vert \langle \varvec{z}(\cdot ,t)\rangle -\langle \varvec{z}^*\rangle \Vert _{L^2}^2+(1+C_1)\Vert \nabla \varvec{z}^*(\cdot ,t)\Vert _{L^2}^2\\&\le \frac{1}{|\Omega |}\Vert \varvec{u}(\cdot ,t)-\varvec{u}^*\Vert _{L^2}^2+(1+C_1)\Vert \nabla \varvec{z}^*(\cdot ,t)\Vert _{L^2}^2\\&\le \frac{\varepsilon ^2}{|\Omega |}+\frac{2(1+C_1)\varepsilon ^2}{\min \{\theta _1,\theta _2\}}. \end{aligned}$$

Combining this inequality with (B.11), we obtain the desired assertion. $\square$

## Computation of the energy

In order to verify (3.24), we first plug $\phi$ and $\psi$ of (3.22) and (3.23) into (3.10). In view of (3.19) and (3.21), we have

C.1 $$\begin{aligned} \begin{aligned}&m_1-\frac{1-d_1/D_1}{L}\int _0^\ell U_1\ dx_1 \\&\quad =m_1-\frac{1-d_1/D_1}{L}\mu _1(\ell )(\ell -\omega _1\tanh (\ell /\omega _1)) =\beta _1\mu _1(\ell )/\gamma _1,\\&m_2-\frac{1-d_2/D_2}{L}\int _\ell ^{L} U_2\ dx_1 \\&\quad =m_2-\mu _2(\ell )\frac{1-d_2/D_2}{L}(L-\ell -\omega _2\tanh ((L-\ell )/\omega _2))=\beta _2\mu _2(\ell )/\gamma _2, \end{aligned} \end{aligned}$$

in the sequel,

$$\begin{aligned}&d_1\Delta U_1-\beta _1U_1+\gamma _1\left( m_1-\frac{1-d_1/D_1}{L}\int _0^\ell U_1\ dx_1\right) =0 \\&\qquad \quad ((x_1, x')\in (0, \ell )\times D),\\&d_2\Delta U_2-\beta _2U_2+\gamma _2\left( m_2-\frac{1-d_2/D_2}{L}\int _\ell ^L U_2\ dx_1\right) =0\\&\qquad \quad ((x_1, x')\in (\ell , L-\ell )\times D). \end{aligned}$$

We set

C.2 $$\begin{aligned} \delta U_i:=\rho _i\mu _i(\ell ), \quad \rho _i:=\frac{\beta _i}{\gamma _i(1-d_i/D_i)+\beta _i}\qquad (i=1,2). \end{aligned}$$

Then, making use of (C.1), we compute

C.3 $$\begin{aligned} \begin{aligned}&m_1-(1-d_1/D_1)\langle \phi \rangle =m_1-(1-d_1/D_1)\left( \frac{1}{L}\int _0^\ell U_1\ dx_1+\delta U_1\right) \\&\quad = \beta _1 \mu _1(\ell )/\gamma _1-(1-d_1/D_1)\mu _1(\ell )\rho _1 \\&\quad = (\beta _1/\gamma _1)\mu _1(\ell )\left( 1-\frac{\gamma _1(1-d_1/D_1)}{\gamma _1(1-d_1/D_1)+\beta _1}\right) \\&\quad = (\beta _1/\gamma _1)\mu _1(\ell )\rho _1, \end{aligned} \end{aligned}$$

and

C.4 $$\begin{aligned} \begin{aligned}&m_2-(1-d_2/D_2)\langle \psi \rangle =m_2-(1-d_2/D_2)\left( \frac{1}{L}\int _\ell ^L U_2\ dx_1+\delta U_2\right) \\&\quad = (\beta _2/\gamma _2)\mu _2(\ell )\rho _2, \end{aligned} \end{aligned}$$

therefore,

$$\begin{aligned}&d_1\Delta \phi -\beta _1\phi -k\psi ^2\phi +\gamma _1\{m_1-(1-d_1/D_1)\langle \phi \rangle \} \\&\quad ={\left\{ \begin{array}{ll} -\beta _1\mu _1(\ell )-k(\rho _2\mu _2(\ell ))^2(U_1+\rho _1\mu _1(\ell )) &{} ((x_1, x')\in (0, \ell )\times D), \\ -k(U_2+\delta U_2)^2\rho _1\mu _1(\ell ) &{} ((x_1, x')\in (\ell , L-\ell )\times D). \end{array}\right. } \end{aligned}$$

Similarly,

$$\begin{aligned}&d_2\Delta \psi -\beta _2\psi -k\phi ^2\psi +\gamma _2\{m_2-(1-d_2/D_2)\langle \psi \rangle \} \\&\quad ={\left\{ \begin{array}{ll} -k(U_1+\delta U_1)^2\rho _2\mu _2(\ell ) &{} ((x_1, x')\in (0, \ell )\times D), \\ -\beta _2\mu _2(\ell )-k(\rho _1\mu _1(\ell ))^2(U_2+\rho _2\mu _2(\ell )) &{} ((x_1, x')\in (\ell , L)\times D). \end{array}\right. } \end{aligned}$$

Hence, it is expected that $(\phi , \psi )$ serves as an approximate solution if $\beta _i~(i=1,2)$ are sufficiently small.

We derive ${{\mathcal {E}}}_s(\phi ,\psi )$ of (3.24). In view of the formulae

$$\begin{aligned} \int (\sinh (x_1/\omega _i))^2\ dx_1&=\frac{\omega _i}{2}\cosh (x_1/\omega _i)\sinh (x_1/\omega _i)-\frac{x_1}{2}, \\ \int (\cosh (x_1/\omega _i))^2\ dx_1&=\frac{\omega _i}{2}\cosh (x_1/\omega _i)\sinh (x_1/\omega _i) + \frac{x_1}{2}, \end{aligned}$$

we have

$$\begin{aligned} \frac{d_1}{2}\int _\Omega |\nabla \phi |^2\ dx&=\frac{|D|d_1}{2} \frac{(\mu _1(\ell ))^2}{\omega _1^2} \int _0^\ell \frac{\sinh ^2(x_1/\omega _1)}{\cosh ^2(\ell /\omega _1)}\ dx_1\\&=\frac{|D|\beta _1}{2}(\mu _1(\ell ))^2 \left( \frac{1}{2}\omega _1\tanh (\ell /\omega _1)-\frac{\ell }{2\cosh ^2(\ell /\omega _1)}\right) , \\ \frac{d_2}{2}\int _\Omega |\nabla \psi |^2\ dx&=\frac{|D|d_2}{2} \frac{(\mu _2(\ell ))^2}{\omega _2^2} \int _\ell ^L \frac{\sinh ^2((L-x_1)/\omega _2)}{\cosh ^2((L-\ell )/\omega _2)}\ dx_1\\&=\frac{|D|\beta _2}{2}(\mu _2(\ell ))^2 \left( \frac{1}{2}\omega _2\tanh ((L-\ell )/\omega _2)\right. \\&\quad \left. -\frac{L-\ell }{2\cosh ^2((L-\ell )/\omega _2)}\right) . \end{aligned}$$

Next we compute

$$\begin{aligned}&\frac{\beta _1}{2}\int _\Omega \phi ^2\ dx =\frac{|D|\beta _1}{2}\left\{ \int _0^\ell (U_1^2 +2(\delta U_1)U_1)dx_1+L(\delta U_1)^2\right\} ,\\&\frac{\beta _2}{2}\int _\Omega \psi ^2\ dx =\frac{|D|\beta _2}{2}\left\{ \int _\ell ^L (U_2^2 +2(\delta U_2)U_2)dx_1+L(\delta U_2)^2\right\} . \end{aligned}$$

We verify

$$\begin{aligned} \int _0^\ell U_1^2 dx_1&=(\mu _1(\ell ))^2 \int _0^\ell \left( 1-2\frac{\cosh (x_1/\omega _1)}{\cosh (\ell /\omega _1)}+ \frac{\cosh ^2(x_1/\omega _1)}{\cosh ^2(\ell /\omega _1)} \right) dx_1\\&= (\mu _1(\ell ))^2 \left\{ \ell -2\omega _1\tanh (\ell /\omega _1)+\frac{\omega _1}{2}\tanh (\ell /\omega _1) +\frac{\ell }{2\cosh ^2(\ell /\omega _1)}\right\} \\&=(\mu _1(\ell ))^2 \left\{ \ell -\frac{3}{2}\omega _1\tanh (\ell /\omega _1) +\frac{\ell }{2\cosh ^2(\ell /\omega _1)}\right\} , \end{aligned}$$

and

$$\begin{aligned}&\int _0^\ell (U_1^2 +2(\delta U_1)U_1)dx_1+L(\delta U_1)^2 \\&\quad =(\mu _1(\ell ))^2\left\{ \ell -\frac{3}{2}\omega _1\tanh (\ell /\omega _1) +\frac{\ell }{2\cosh ^2(\ell /\omega _1)}\right. \\&\qquad \left. + 2\rho _1(\ell -\omega _1\tanh (\ell /\omega _1))+L\rho _1^2\right\} . \end{aligned}$$

In the sequel we have

C.5 $$\begin{aligned} \begin{aligned}&\frac{d_1}{2}\int _{\Omega }|\nabla \phi |^2\ dx+\frac{\beta _1}{2}\int _{\Omega }\phi ^2\ dx \\&\quad =\frac{|D|\beta _1}{2}\mu _1(\ell )^2 \{\ell (1+\rho _1)^2 +(L-\ell )\rho _1^2-(1+2\rho _1)\omega _1\tanh (\ell /\omega _1)\}. \end{aligned} \end{aligned}$$

By the similar computation we obtain

$$\begin{aligned}&\int _\ell ^L U_2^2 dx_1 =(\mu _2(\ell ))^2 \int _\ell ^L \left( 1-2\frac{\cosh ((L-x_1)/\omega _2)}{\cosh ((L-\ell )/\omega _2)}+ \frac{\cosh ^2((L-x_1)/\omega _2)}{\cosh ^2((L-\ell )/\omega _2)} \right) dx_1 \\&\quad =(\mu _2(\ell ))^2 \left\{ L-\ell -\frac{3}{2}\omega _2\tanh ((L-\ell )/\omega _2) +\frac{L-\ell }{2\cosh ^2((L-\ell )/\omega _2)}\right\} , \\&\quad \quad \int _\ell ^L(U_2^2+2(\delta U_2) U_2)dx_1+L(\delta U_2)^2\\&\quad =(\mu _2(\ell ))^2 \left\{ L-\ell -\frac{3}{2}\omega _2\tanh ((L-\ell )/\omega _2) +\frac{L-\ell }{2\cosh ^2((L-\ell )/\omega _2)} \right. \\&\quad \quad \left. \qquad + 2\rho _2(L-\ell -\omega _2\tanh ((L-\ell )/\omega _2))+L\rho _2^2\right\} , \end{aligned}$$

in the sequel,

C.6 $$\begin{aligned} \begin{aligned}&\frac{d_2}{2}\int _{\Omega }|\nabla \psi |^2\ dx+\frac{\beta _2}{2}\int _{\Omega }\psi ^2\ dx \\&\quad =\frac{|D|\beta _2}{2}\mu _2(\ell )^2 \{(L-\ell )(1+\rho _2)^2+\ell \rho _2^2-(1+2\rho _2)\omega _2\tanh ((L-\ell )/\omega _2)\}. \end{aligned} \end{aligned}$$

On the other hand, making use of (C.3) and (C.4), we obtain

C.7 $$\begin{aligned} \begin{aligned}&\frac{\gamma _1|\Omega |}{2(1-d_1/D_1)}(m_1-(1-d_1/D_1)\langle \phi \rangle )^2 =\frac{|\Omega |}{2(1-d_1/D_1)}\frac{\beta _1^2}{\gamma _1}(\mu _1(\ell ))^2\rho _1^2, \\&\frac{\gamma _2|\Omega |}{2(1-d_1/D_1)}(m_2-(1-d_2/D_2)\langle \psi \rangle )^2 =\frac{|\Omega |}{2(1-d_2/D_2)}\frac{\beta _2^2}{\gamma _2}(\mu _2(\ell ))^2\rho _2^2.\\ \end{aligned} \end{aligned}$$

In addition,

C.8 $$\begin{aligned} \begin{aligned}&\int _0^L \phi ^2\psi ^2\, dx=|D|\left( \int _0^\ell (U_1+\delta U_1)^2(\delta U_2)^2\ dx_1 +\int _\ell ^L(\delta U_1)^2(U_2+\delta U_2)^2\ dx_1\right) \\&\quad =|D| \left( (\mu _2(\ell )\rho _2)^2 \int _0^\ell (U_1+\delta U_1)^2 dx_1 +(\mu _1(\ell )\rho _1)^2\int _\ell ^{L}(U_2+\delta U_2)^2 dx_1\right) \\&\quad =|D|(\mu _1(\ell )\mu _2(\ell ))^2 \left\{ \rho _2^2 \left( \ell (1+\rho _1)^2\right. \right. \\&\qquad \left. -(\frac{3}{2}+2\rho _1)\omega _1\tanh (\ell /\omega _1) +\frac{\ell }{2\cosh ^2(\ell /\omega _1)} \right) \\&\quad +\rho _1^2\left( (L-\ell )(1+\rho _2)^2-(\frac{3}{2}+2\rho _2)\omega _2\tanh ((L-\ell )/\omega _2)\right. \\&\quad \left. \left. +\frac{L-\ell }{2\cosh ^2((L-\ell )/\omega _2)} \right) \right\} \end{aligned} \end{aligned}$$

Combining (C.5), (C.6), (C.7) and (C.8), we obtain (3.24).
